# Identified Three Interferon Induced Proteins as Novel Biomarkers of Human Ischemic Cardiomyopathy

**DOI:** 10.3390/ijms222313116

**Published:** 2021-12-04

**Authors:** Cheng Chen, Jiao Tian, Zhicheng He, Wenyong Xiong, Yingying He, Shubai Liu

**Affiliations:** 1 State Key Laboratory of Phytochemistry and Plant Resources in West China, Kunming Institute of Botany, Chinese Academy of Sciences, Kunming 650201, China; Chencheng@mail.kib.ac.cn (C.C.); tianjiao@mail.kib.ac.cn (J.T.); hezhicheng@mail.kib.ac.cn (Z.H.); wenyong.xiong@mail.kib.ac.cn (W.X.); 2 University of Chinese Academy of Sciences, Beijing 100049, China; 3 School of Life Sciences, Yunnan University, Kunming 650091, China; 4 School of Chemical Science & Technology, Yunnan University, Kunming 650091, China

**Keywords:** ischemic cardiomyopathy, heart failure, WGCNA, interferon stimulated genes, IFIT2/3

## Abstract

Ischemic cardiomyopathy is the most frequent type of heart disease, and it is a major cause of myocardial infarction (MI) and heart failure (HF), both of which require expensive medical treatment. Precise biomarkers and therapy targets must be developed to enhance improve diagnosis and treatment. In this study, the transcriptional profiles of 313 patients’ left ventricle biopsies were obtained from the PubMed database, and functional genes that were significantly related to ischemic cardiomyopathy were screened using the Weighted Gene Co-Expression Network Analysis and protein–protein interaction (PPI) networks enrichment analysis. The rat myocardial infarction model was developed to validate these findings. Finally, the putative signature genes were blasted through the common Cardiovascular Disease Knowledge Portal to explore if they were associated with cardiovascular disorder. Three interferon stimulated genes (IFIT2, IFIT3 and IFI44L), as well as key pathways, have been identified as potential biomarkers and therapeutic targets for ischemic cardiomyopathy, and their alternations or mutations have been proven to be strongly linked to cardiac disorders. These novel signature genes could be utilized as bio-markers or potential therapeutic objectives in precise clinical diagnosis and treatment of ischemic cardiomyopathy.

## 1. Introduction

Ischemic cardiomyopathy (ISCM) is a symptom in which blood flow to heart is reduced or stopped, resulting in heart muscle injury [1]. This persistent ISCM is the most prevalent cause of heart failure (HF) and a leading cause of death worldwide. As a result of the global pandemic, up to 26 million people were affected by cardiac insufficiency, costing the global health system more than $30 billion [2,3]. Furthermore, mortality in patients with cardiac disorders is above 50% within five years [4,5].

Interferons (IFNs) are cytokines secreted by mammalian cells in response to lethal viral infection. IFNs can engage a serial of signal transduction cascades and induce hundreds of interferon-stimulated genes (ISGs), some of which have been identified with functions of specific antiviral and others that are unknown [6]. IFIT (IFN-induced proteins with Tetratricopeptide Repeat (TPR) motifs) family proteins bind eiF3C or eiF3E to inhibit translation initiation pathogens, including IFIT1/1B, IFIT2, IFIT3 and IFIT5 in human and six members in murine (IFIT1/1B/1C, IFIT2, IFIT3/3B), and target viral protein production through a variety of mechanisms [7]. The TPR motif is a degenerate thirty-four amino acid residue repeat unit that is important in protein–protein interaction and large protein complex assembly [7,8]. Multiple TPR domains is thought to confer a wide range of effects on cellular and viral functions, including transcription and translation regulation, anti-proliferative effects, and negative regulation of host inflammatory responses as well as antiviral response and antiviral response [8]. Overexpression of IFIT2 causes caspase-3 activation and disrupts plasma membrane asymmetry, both of which are hallmarks of apoptotic cell death [9]. IFIT2-mediated apoptosis is independent of DNA damage responses [10]. Instead, IFIT2 acts through the mitochondrial pathway, where the balance of pro- and anti-apoptotic Bcl-2 family proteins regulates the permeability of the outer mitochondrial membrane [11]. However, little is known about the exactly role of IFITs in ISCM.

Early clinical investigations demonstrated that heart failure patients with dysregulated gene expression profile, which has normal somatic genetics background [12]. Recently, to improve the patient’s treatment and healthcare management outcomes, it is the trend to discover applicable disease signature genes or biomarkers for early diagnostics through analyzing the genetic disorder and expression profiling of heart failure [13,14]. Among multiple computerization methodologies, the Weighted Gene Co-Expression Network Analysis (WGCNA) is considered as one of the most useful approaches to discover gene co-expression network based functional feature through gene expression profiling analysis [15]. Furthermore, WGCNA has been widely applied to screen the novel biomarkers or therapeutic targets for cancer early diagnostics and treatment, such as hepatocellular carcinoma [16] and lung cancer [17].

In this study, WGCNA was used to analyze gene expression patterns of roughly 300 clinical ISCM patients to discover the significance genes associated with disease. Co-expression and protein–protein interaction (PPI) networks enrichment analyses were used to discover the hallmark genes and critical pathways. The rat MI model was used to validate the screened signature genes. Finally, the cardiovascular disease data portal was used to explore the potential biomarkers.

## 2. Results

### 2.1. Identify the Module and Significance Genes Related to ISCM

**Figure 1:**
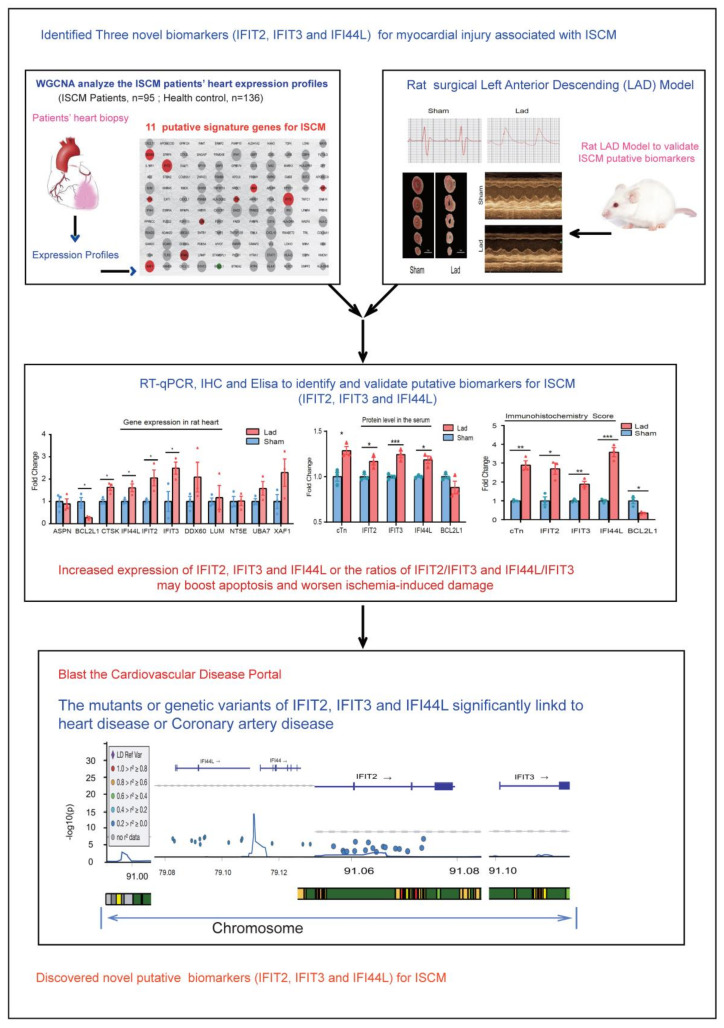
Overview workflow of the data analysis and experimental design. *, *p* < 0.05; **, *p* < 0.01; ***, *p* < 0.001.

**Figure 2:**
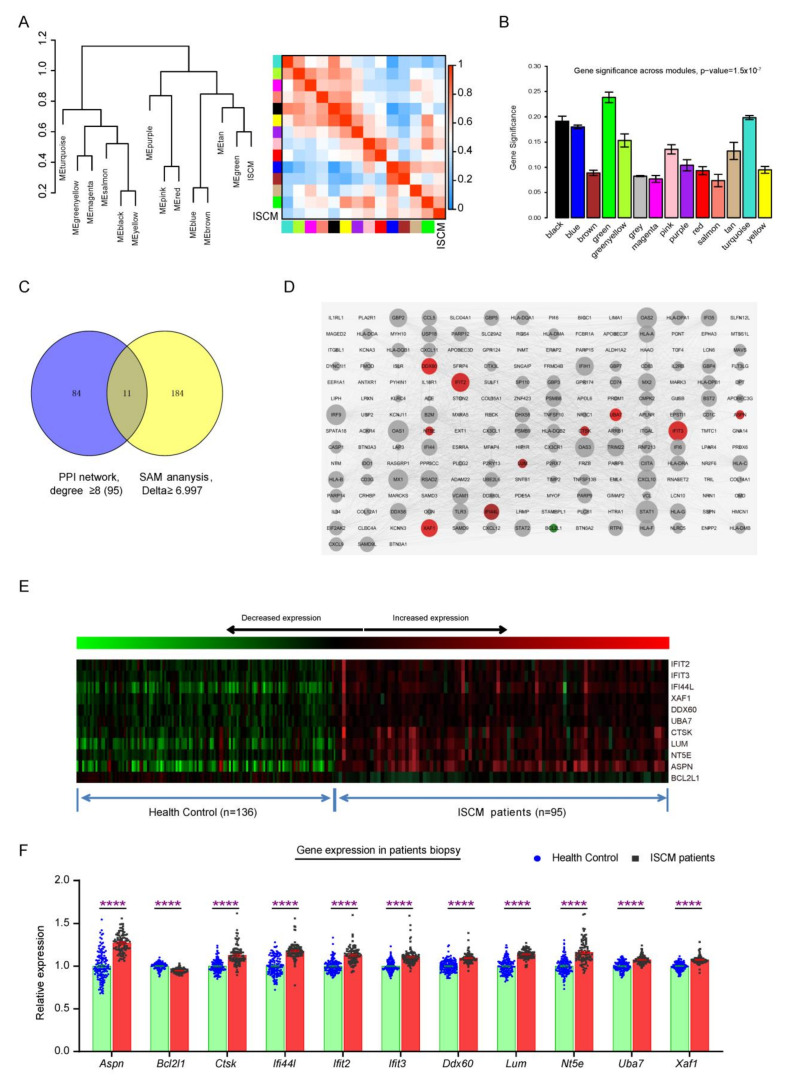
Identified the signature genes significantly associated with ISCM. (**A**) Clustering dendrogram of the eigengenes and adjacency with ISCM dissimilarity based on topological overlap. The left panel shows a hierarchical clustering dendrogram of the eigengenes in which the dissimilarity of eigengenes. The heatmap in right panel shows the eigengenes adjacency. (**B**) The gene significance of all modules identified correlated with ISCM. The module membership vs. gene significance of green module that the most significantly correlated to ISCM. (**C**) The hub genes indicated by Venn diagram for SAM analysis and PPI network. (**D**) The PPI network of genes contained with GS-module by STRING database. Their intersection is defined as hub gene. The node size is designed with the degree value, the low value to small sizes. The high expression gene is red node and low expression is green. The gray nodes indicate that it is not a differentially expressed gene. The signature genes expression in the whole transcription profiling was plotted by heatmap (**E**) and histogram (**F**). The data including health control patients (136 samples) and ISCM patients (95 samples) and were expressed as the means ± SEM (**** *p* < 0.0001).

The whole transcription profiles of 231 patient’s biopsy were used in this study, as shown in the workflow (Figure 1), including health (136 cases) and ISCM patients (95 cases, Supplementary Table S1). The WGCNA and the hierarchical clustering were used to identify distinct modules related to ISCM. The significant modules that correlated to the ISCM feature were identified by calculating and comparing the module memberships (MM) correlation and Genes Significance (GS). Combined with the Eigengene dendrogram analysis and the module–trait relationship correlation results, the green module was clustered with ISCM tightly (Figure 2A, Supplementary Table S2) and the contained genes has the strongest correlation with ISCM status (Figure 2A,B). The scatter plot of MM was plotted against the GS in each significant module, with each point representing a gene contained in a module (Supplementary Figure S2).

### 2.2. Identification of Signature Genes Associated with ISCM

The significance genes contained in green module were put into STRING Database to explore the interaction nodes. The PPI network consisted of 199 proteins (nodes) and 3240 directed edges, where the node size indicated the degree value and the edge weight represented the confidence in the predicted direction. Genes with highest degree of connectivity are located at the core of PPI network. The 95 candidate genes were identified by using the PPI network method and connective degree over eight filtering (Supplementary Table S3). SAM analysis, on the other hand, identified 195 differentially expressed genes. (Supplementary Table S4). Combined the results of PPI network and SAM analysis, there are 11 signature genes were chosen to further investigation (Figure 2C, Supplementary Table S6). These signature genes were visualized and labelled using Cytoscape software and integrated into PPI networks. Ten genes (*IFIT2*, *IFIT3*, *XAF1*, *DDX60*, *IFI44L*, *UBA7*, *CTSK*, *LUM*, *NT5E*, *ASPN*) were up-regulated (red color), while *BCL2L1* was down-regulated (green) (Figure 2D). The signature genes were also visualized using heatmap (Figure 2E) and histogram (Figure 2F). These signature genes are thought to play dominant role and work as common key regulatory nodes in the progress of ISCM.

### 2.3. Functions and Pathways Enrichment Analysis

**Figure 3:**
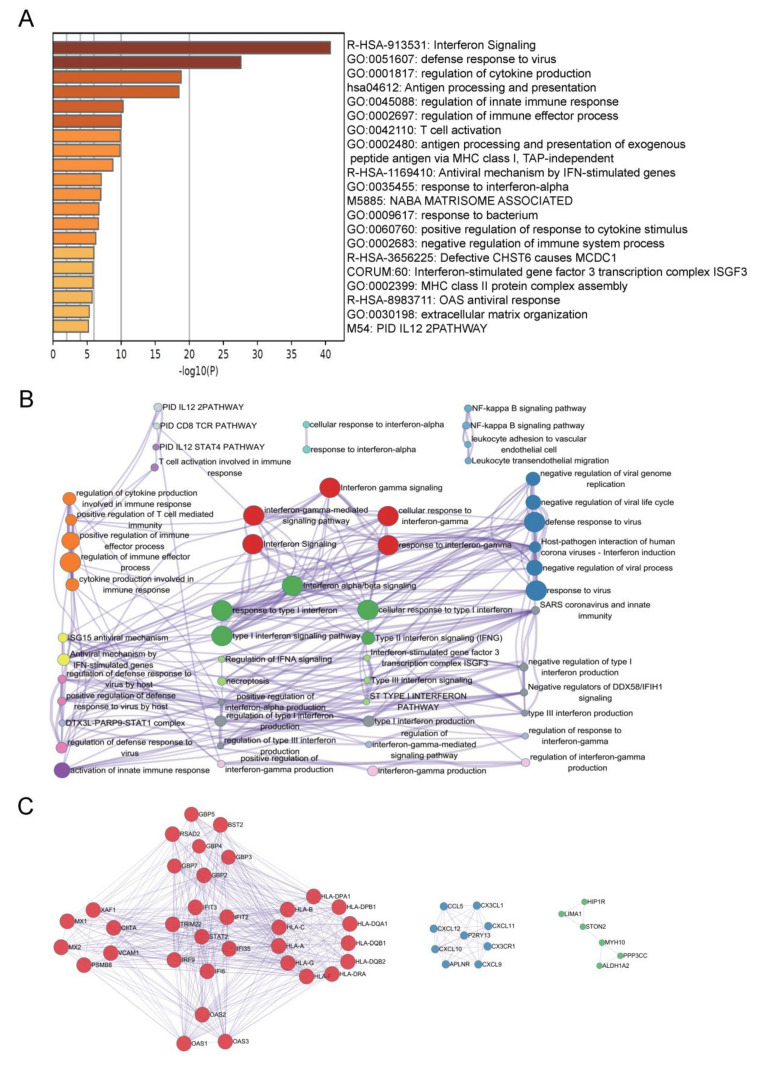
Function and pathway enrichment analysis. (**A**) The heatmap shows the top 20 clusters of enriched sets. (**B**) Representative Molecular Complex Detection (MCODE) network node involving by ISCM significant associated genes. (**C**) Representative MCODE network node showing the key signaling pathways involved by ISCM-associated significant genes. Metascape analysis. A network of enriched sets colored by ID. Threshold: 0.3 kappa score; similarity score > 0.3. Heatmap colored by *p*-values.

Notably, the significance genes contained in green module, which is the most strongly significantly correlated with ISCM (268 genes), were investigated biological functions by Gene ontology (GO) enrichment and Kyoto Encyclopedia of Genes and Genomes (KEGG) pathway analyses. The top 20 enrichment signaling pathways were summarized, including interferon signaling, defense response to virus, regulation of cytokine production and regulation of innate immune response, etc. (Figure 3A, Supplementary Table S5). A subset of enriched terms was selected and presented as a network graph to further discover the connections that exist between terms, which were determined by the term with the best *p*-value (Figure 3B). The top three PPI MCODE components were labeled by constative genes (Figure 3C), including cytokine-mediated signaling pathway, interferon signaling and positive regulation of cytokine production.

### 2.4. LAD Rat Model Validated the Signature Genes Expression

**Figure 4:**
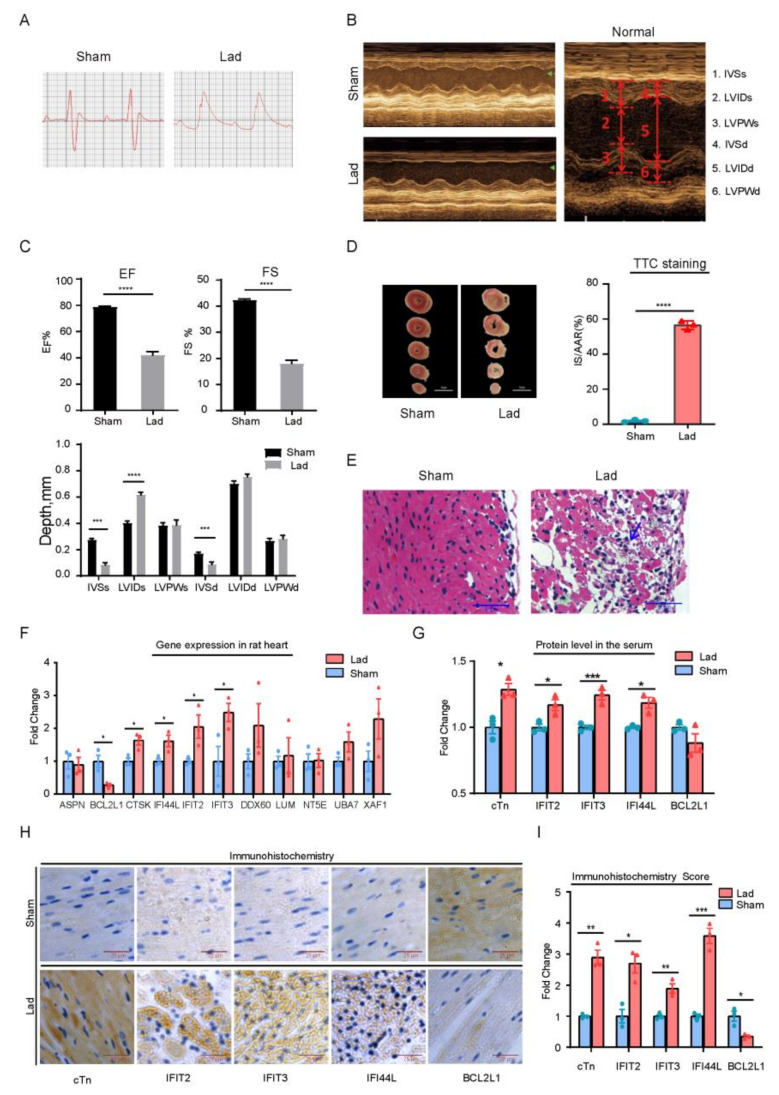
The evaluation of cardiac function and hub genes expression in heart. (**A**) After ligation, the S–T segment of the electrocardiogram was raised with arch back. (**B**) The typical echocardiographic analysis of different groups, including the Normal, Sham, and myocardial infarction (Lad). (**C**) Evaluate the cardiac function. LVEF: LV ejection fraction; LVFS: LV fractional shortening; IVSs, interventricular septal thickness in systole; LVIDs: LV internal diaMeter in systole; LVPWs: LV posterior wall thickness in systole; IVSd: interventricular septal thickness in diastole; LVIDd: LV internal dimension-diastole; LVPWd: LV posterior wall thickness in diastole. The TTC staining (**D**) and hematoxylin and eosin (**E**). The arrows indicate that the cardiomyocytes were incomplete and the nuclear distribution was not clear. Lad group is the ligation of left anterior descending branch for 24 h. (**F**) The mRNA level in LV. (**G**) Elisa assay tested the concentration of cTn, BCL2L1, IFIT2, IFIT3 and IFI44L in rat serum. (**H**,**I**) Immunohistochemical analysis and quantitative score of BCL2L1, IFIT2, IFIT3 IFI44L and cTn in cardiac tissues. The cTn was used as a reference protein. Results were expressed as the means ± SEM, unpaired *t*-test was used to analysis the difference between the two groups (Sham group: *n* = 3; Lad group *n* = 3; * *p* < 0.05, ** *p* < 0.01, *** *p* < 0.001 and **** *p* < 0.0001 vs. Sham group).

The cardiac function was measured by echocardiography after 1 day of continuous ischemia to validate the model’s success. The value of left ventricular ejection fraction (EF) and fractional shortening (FS) were used as markers to monitor the cardiac function. The left ventricle (LV) was clearly dilated and morphologically abnormal, and the motion of anterior, lateral, and anteroseptal walls was reduced or even absent in the MI rats, according to echocardiography (Figure 4A,B). The LVEF was significantly reduced from 78.84% to 42.14% (*n* = 10, *p* < 0.0001) and FS significantly reduced from 42.38% to 18.03% (*n* = 10, *p* < 0.0001) (Figure 4C).

The MI group’s infraction size was detected by TTC staining. The relative region of infarction is 56.53 ± 1.420 (*n* = 3, *p* < 0.0001) (Figure 4C). The cardiomyocytes in the sham group were intact, neatly arranged and showed no necrosis according to HE staining. Cardiomyocytes in MI group are incomplete, and the cell nuclear distribution is unclear (Figure 4E, Lad). According to the statistics, an infarction had occurred. The rat myocardial ischemia model had been successfully established.

RT-qPCR was used to evaluate the expression of these signature genes in the LV (Supplementary Table S7). Five signature genes were found to be significantly expressed in LAD groups, according to the findings. *BCL2L1* was down-regulated, while *CTSK*, IFIT2, IFIT3 and *IFI44L* were up-regulated (Figure 4F). In heart tissue, in situ immunohistochemistry revealed that IFI44L, IFIT2 and IFIT3 were up-regulated, while BCL2L1 was down-regulated. This data was consistent with the qPCR results (Figure 4H,I). The protein levels in the rat serum were measured by ELISA Method to see if these genes may be used as diagnostic biomarkers. IFIT2, ITIT3 and IFI44L were significantly higher in the Lad group, while BCL2L1 was significantly lower (Figure 4G). Together, it is strongly suggested that these genes (*IFIT2,* IFIT3, IFI44L and *BCL2L1*) would be used as valuable biomarkers for ISCM.

### 2.5. Metoprolol Administration following MI Protect 

**Figure 5:**
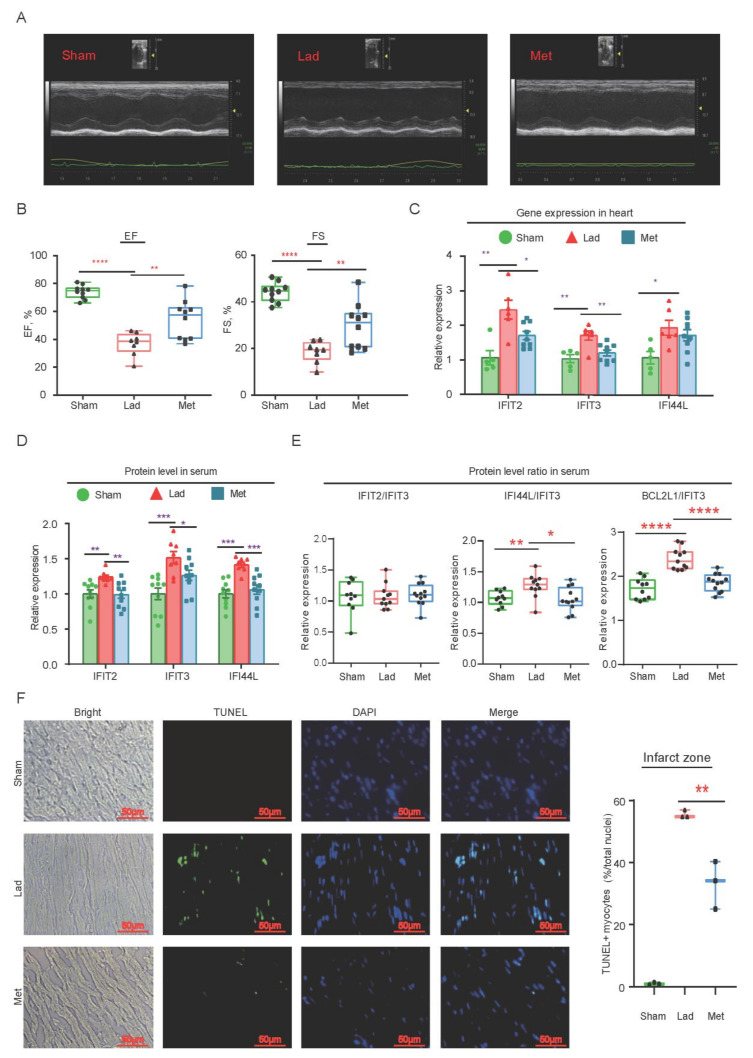
Cardiac function evaluation in rats after continuous administration Metoprolol. (**A**) The echocardiographic analysis of different groups, including Sham, myocardial infarction (Lad) and Met group. (**B**) The average of EF and FS in the Met group were significantly higher than Lad group. Sham group: *n* = 10; Lad group *n* = 8; Met group: *n* = 10. (**C**) The RT-qPCR to test gene expression in left ventricle (Sham group: *n* = 5; Lad group: *n* = 6; Met group: *n* = 8). (**D**) The proteins in MI rat serum were detected by ELISA (Sham group: *n* = 10; Lad group: *n* = 8; Met group: *n* = 10). (**E**) The ratios of proteins. The proportion of IFIT2/IFIT3 in sham group, Lad group and Met group were 1.066 ± 0.08, 1.076 ± 0.05 and 1.104 ± 0.05, respectively. The ratio of BCL2L1/IFIT3 was significantly increased in LAD group (2.399 ± 0.07), compared with Sham group (1.715 ± 0.07) and MET group (1.859 ± 0.05). The proportion of IFI44L/IFIT2 was similar with BCL2L1/IFIT3 (Sham group, 1.07 ± 0.04; LAD group, 1.27 ± 0.06; MET group, 1.055 ± 0.05). Sham group: *n* = 10; Lad group: *n* = 11; Met group: *n* = 12. (**F**) Cardiomyocyte’s apoptosis in infract zone. DAPI channel showed blue fluorescence and TUNEL-positive cardiomyocytes indicated green fluorescence and the merged channel was DAPI and TUNEL. LAD group, 55.50 ± 0.72; MET group, 33.15 ± 4.45. *p* = 0.0077, *n* = 3. All results were expressed as the means ± SEM and a dependent *t*-test would be used. * Represents *p* < 0.05; ** represents *p* < 0.01; *** represents *p* < 0.001; **** represents *p* < 0.0001.

Following the successful creation of MI model, the positive control group (Met, *n* = 12) received Metoprolol (2 mg/kg, three times per day) intravenously for 3 days. The average of IVSd, IVSs, LVIDs, EF and FS in the MI group were significantly lower than Met group, as measured by echocardiography (Figure 5A,B and Supplementary Figure S3c–h). Metoprolol significantly reduced morphologically abnormalities of LV and anterior motion caused by ischemia when compared to the MI group. These results validated that the Metoprolol protected cardiac function in the MI rat.

The RT-qPCR result showed that the level of *IFIT2,* *IFIT3* and *IFI44L* in the heart was significantly lower than the MI group, but still higher than the sham group after 3 days of Metoprolol administration (Figure 5C). The serum level of IFIT2, ITIT3 and IFI44L were significantly lower than MI rats (Figure 5D). In addition, in a rat MI model, the ratios of IFIT2/IFIT3, IFI44L/IFIT3 and BCL2L1/IFIT3 were significantly decreased, which was associated with Metoprolol’s cardiac protection (Figure 5E). In the ischemic heart, Metoprolol, a cardiac protectant drug, has been suggested to reduce the expression of these hallmark genes. It is the possible that *IFIT2*, *IFIT3* and *IFI44L* will be identified as potential pathological biomarkers for ISCM.

### 2.6. Cardiomyocyte Apoptosis in Infract Zone

Apoptosis of cardiomyocyte was also detected. The positive TUNEL signal of cardiomyocytes in LAD group (percentage of total nuclei) was higher than Met group (Figure 5F). These results indicated that Metoprolol could protect heart by through reducing apoptosis following ischemic event. Increased expression of IFIT2, IFIT3 and IFI44L, as well as the ratios of IFIT2/IFIT3 and IFI44L/IFIT3, may boost apoptosis and aggravate ischemia-induced damage.

### 2.7. Blast Signature Genes in the Cardiovascular Disease Portal 

**Figure 6:**
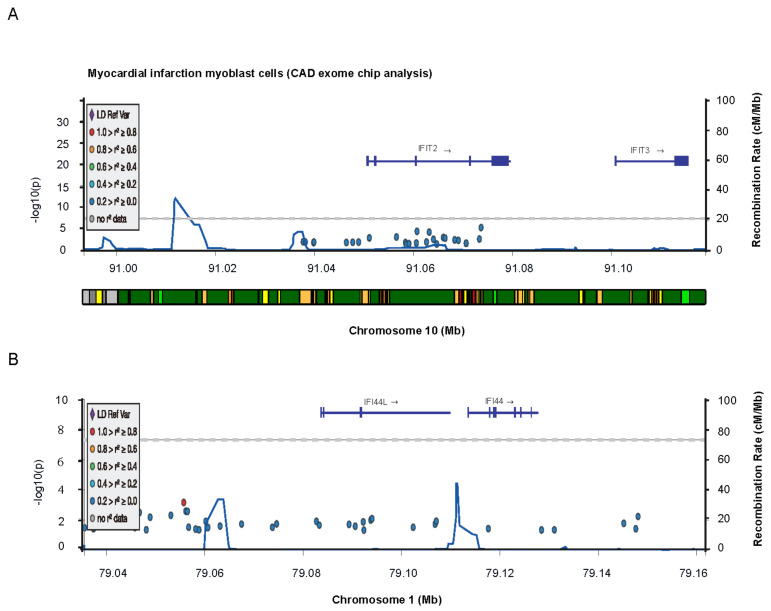
Blast signature genes in the Cardiovascular Disease Knowledge Portal Project Database. The GEnomic region Miner (GEM) module explore target gene variant associations, epigenomic annotations, and credible sets across chromosome. Ifit2 and Ifit3 are located on chromosome 10 (**A**,**B**) and Ifi44l is located on chromosome 1(B).

*IFIT2, IFIT3* and *IFI44L* are significantly related with myocardial infarction and coronary artery disease, according to Cardiovascular Disease Knowledge Portal Project Database (cvd.hugeamp.org). *IFIT2* and *IFIT3* are located on chromosome 10 (Figure 6A,B) and *IFI44L* is located on chromosome 1 (Figure 6B). The most relevant genetic variants of *IFIT2* and *IFIT3* linked to coronary artery disease are extensively summarized (Supplementary Table S8). It is significant evidence that that three signature genes (*IFIT2*, *IFIT3* and *IFI44L*) are linked to heart disease and used as potential biomarker for ISCM. In order to develop novel therapy strategies for ISCM, the regulatory mechanisms of these disease signature genes will be thoroughly investigated in the future.

## 3. Discussion

In this study, a straightforward research strategy was applied to discover the potential signature gene that significantly associated with ISCM. First of all, we screened and filtered the whole transcription profile of ISCM patient’s in the GEO data reservoir, then extracted the transcription profile data for subsequent bioinformatics analysis using WGCNA, which is considered as one of the most useful approaches to discover gene co-expression network based functional features through gene expression profiling analysis [15] and widely applied to screen the novel biomarkers or therapeutic targets for cancer early diagnostics and treatment [16,17]. Next, the identified the significance genes were taken to perform the functions and signaling pathway enrichment analysis through Metascape. The Interferon Signaling was identified as the most significantly enriched pathway among the top twenty significant pathways, and the majority of pathways were involved in the regulation of innate immune response. The Interferon signaling pathway and innate immune response related signaling pathway are strongly suggested to play important role in ISCM. Eleven potential signature genes were identified using the SAM analysis and protein–protein interaction (PPI) networks analysis. A rat MI model that ligated the left anterior descending (LAD) coronary artery to induce myocardial infarction [18] was used as an alternative validation for these signature genes, according to the clinical biopsy limited accession. Four of eleven signature genes were significantly changed and matched the expression pattern identified in the transcription profile of ISCM patient’s biopsy, which were tested by qPCR, IHC and Elisa assay in a rat MI model. Their protein levels in serum and gene expression levels in the rat heart both decreased significantly after being treated with Met in a rat MI model. Furthermore, three potential signature genes (*IFIT2*, *IFIT3* and *IFI44L*) were identified in cardiovascular disease knowledge portal. The genomic variants or mutations of three signature genes were found to be significantly associated with cardiovascular disease (Supplementary Table S8). Three signature genes (*IFIT2*, *IFIT3* and *IFI44L*) have been identified as potential biomarkers for ISCM.

Myocardial infarction (MI) causes massive synchronous cell death in the heart [1]. The leaking of necrotic cardiac myocytes activates damage-associated molecular patterns (DAMPs) via pattern recognition receptors (PRRs) of the innate immune system (e.g., macrophages and dendritic cells), triggering inflammatory response [19]. The activating cytotoxic T cells and transcription factors of interferon regulatory factors could reverse target genes in the heart that encode pro-inflammatory cytokines and interferons [20]. IFNs activate IFIT family members and engage numerous signal transduction cascades to regulate cell-intrinsic and cell-extrinsic immune responses, minimizing immune-mediated damage and cardiac remodeling [21]. Depending on cell type and tissue types, IFIT family proteins execute multiple complex cellular functions. The top different expressed genes in the cardiovascular disease or MI were identified as *IFIT2* and *IFIT3* [19]. IFIT 2/3 was thought to be secreted from classical macrophages or alternative macrophages in the infarcted area of heart, protecting or promoting the reverse healing of heart tissue [19]. FIT family proteins were found to have a protective role in several cell types, including cardiomyocytes (both in HL-1 cells and in primary isolates), peritoneal macrophages, and cardiac fibroblasts, when mice were infected with CVB3. In the heart, IFIT1/3 has a protective role against CVB3 infection. The IFITKO mice, which lack all of the IFIT family genes (IFIT1, IFIT2, IFIT3, IFIT1B, IFIT1C, AND IFIT3B), lost the protective effect, and CVB3 titer increased [22]. Human IFIT2/3 binds and inhibits stimulator of IFN genes STING, a mitochondrial adaptor protein that recruits TANK-binding kinase 1 (TBK1) and IRF3 to a complex with mitochondrial antiviral signaling protein (MAVS), resulting in the downstream induction of IFN β expression in response to viral RNA or DNA56. *IFIT2/3* was reported to associate with the mediator activation of IRF3 and NF-Κ*b* [9,23]. IFNs-induced protein 44-like (IFI44L) was identified as negatively modulator for innate immune response induced by virus infection, which interacts with FKBP5 to decrease the phosphorylation of IRF-3 and NF-B mediated by IKK and IKK, respectively [24]. 

In our study, overexpression of IFIT2/3 were seen in clinical ISCM patient’s biopsies, as well as in heart tissue and serum of rat MI model. The IFIT2/3 level in heart tissue and serum were significantly lower in the Met-treated group than MI LAD group. It is suggested that the level of IFIT2/3 reflect the improvement of cardiac functions in MI patients. Inhibiting IFIT2 degradation by proteasome resulted in perinuclear aggregation and promoted apoptosis [10]. IFIT2 induced apoptosis by balancing pro- and anti-apoptotic Bcl-2 family proteins, which altered the permeability of the mitochondrial membrane [9]. Co-expression of IFIT3 inhibited IFIT2-dependent apoptotic cell death [9]. IFIT2 and IFIT3 could form a protein complex in the cell, IFIT3 negatively regulated the apoptotic effects of IFIT2 [25], and whether or not protect the cell dependent on the ratio of IFIT2: IFIT3 association. 

Furthermore, our results indicated that the IFITs genes (IFIT2, IFIT3 and IFI*44L*) were up-regulated expression in the LAD groups’ rat heart tissue, whereas BCL2L1 was down-regulated, as tested by qPCR and in situ immunohistochemistry. BCL2L1 is a critical anti-apoptosis protein and down-expression will pro-apoptosis [26]. It suggests that MI causes cardiomyocytes apoptosis, and that overexpression of IFIT2 and IFIT3 may attenuate the ventricular damage and improve the cardiac dysfunction and survival. The new direction in future research will be to explore the protecting role and mechanism of IFIT2/3 in post-MI cardio-protection.

There are some limitations in this study. Firstly, it was hard to track these samples’ pathological features with expression profiles and validate these potential biomarkers against the original patient’s pathological feature due to a lack of clinical patient samples and more precise clinical information. Second, the discovered individual GO pathways and biomarkers were not deeply investigated further due to the nature of bioinformatics analysis. Although these genes have been validated as being significantly associated with ISCM features in animal models, more clinic patient plasma samples are needed to confirm these possible biomarkers. In the future, it is necessary and logical to investigate the function and mechanism of these potential signature genes for ISCM using a target gene knockout animal model and cardiac myocytes.

In summary, this study contributes to our understanding of potential novel key regulatory biomarkers or signature genes for ISCM. IFIT*2,* IFIT3 and IFI*44L* have been identified as potential clinical therapy targets.

## 4. Materials and Methods

### 4.1. Study Design and Patient’s Data Involvement

The aim of this study was to discover the novel putative biomarkers for ischemic cardiomyopathy and was reviewed by our institute research ethic board. The design of study has been demonstrated (Figure 1) and the transcription data of cardiomyopathy patients’ biopsy and healthy normal donors download from NCBI GEO. The patient’s grouping and detailed information were described in the original data annotation (GSE57338). In this study, we leveraged the original expression data from NCBI and validated the research discovered putative biomarkers in rat animal model. The protocols of this study were reviewed and approved by the ethic Committee of Kunming Institute of Botany.

### 4.2. Preprocessing of Clinical Patient Samples and Gene Expression Data

These samples and data were generated by Dr. Michael Patrick Morley, Penn Cardiovascular Institute, Perelman School of Medicine at the University of Pennsylvania, Heart Failure and Healthy individuals [27]. The human left ventricle samples were collected heart-failure patients’ biopsy (e.g., idiopathic dilated cardiomyopathy and ischemic cardiomyopathy) and from “healthy normal” organ donors, as described in the annotation, to establish cardiac transcription profiles of heart failure. The gene expression data used in this paper was obtained from the Gene Expression Omnibus (GEO) database in NCBI with tracking number GSE57338, and the transcriptional profiles of 313 patient’s left ventricle biopsies were measured by the Affymetrix Human Gene 1.1 ST Array (platform GPL11532) (https://www.ncbi.nlm.nih.gov/geo/query/acc.cgi?acc=GSE57338, accessed on 1 January 2015). The datasets contained 28,000 target genes for further analysis (Supplementary Table S1), which contains total of 33,297 probes that correspond to measuring changes in transcriptional profiles that are correlated with the physiologic profile of heart-failure hearts. Each gene expression value was normalized and transformed using log2. The biopsy samples were represented three subgroups, including normal hearts as control (Health, *n* = 136), and idiopathic cardiomyopathy (IDCM, *n* = 82), ischemic cardiomyopathy (ISCM, *n* = 95), respectively. The quality of microarray was evaluated using sample clustering based on the distance between different samples in Pearson’s correlation matrices, and a height cut of 90 was chosen to identify potential microarray outliers. Two samples (GSM1379815/1380018) of ischemic cardiomyopathy were detected as outliers and ignored in the subsequent analysis. 

### 4.3. Construction of Weighted Gene Co-Expression Network 

The WGCNA package of R (version 1.63) was download and set up by following the protocol described previously [28]. The WGCNA package was used for performing various functions in weighted correlation network analysis, including constructing network, detecting module, calculating topological properties, simulating data, visualization, and interfacing with external software [28]. First of all, data has been checked to exclude the sample with excessive missing values and identification of outlier samples. After the data were preprocessed, the principal component analysis (PCA) was applied to double check the data quality. The heart failure and health samples were separated in the PCA plot (Supplementary Figure S1a), and the hierarchical clustering on the samples was performed to detect potential outliers. The total 231 samples were used for next step analysis (Supplementary Figure S1a). The soft threshold β = 7 was chose to construct the co-expression network as the R^2^ reached the peak for the first time when β = 7. The plot of log10(*p*(k)) versus log10(k) indicated that the network was close to a scale-free network by using β = 7, where k was the whole network connectivity and *p*(k) was the corresponding frequency distribution. When β = 7, R^2^ is 0.98, ensuring that the network was close to the scale-free network. After the soft thresholding power β was determined, the Topological Overlap Matrix (TOM) and dissTOM = 1 − TOM were obtained. After the modules were identified, the *t*-test was used to calculate the significant *p*-value of candidate genes, and the gene significance (GS) was defined as mediated *p*-value of each gene (GS = lgP). Then, the module significance (MS) was defined as the average GS of all the genes involved in the module. The cut-off significant standard was set up as *p*-value lower than 0.05. In general, the module with the highest MS among all the selected modules will be considered as the one associated with disease. In addition, it was also calculated the relevance between the different type (idiopathic cardiomyopathy, ischemic cardiomyopathy) of modules and cardiomyopathies phenotypes to identify the most relevant module. In the WGCNA, the module membership (MM): MM(i) = cor (xi, ME) is defined to measure the importance of the gene within the module. The greater absolute value of MM(i), the gene i is more important in the module. The Genes Significance (GS) in the module is highly correlated with MM and the most important element to discover the significant module, indicating that genes in the module are significantly associated with cardiomyopathies feature. The hierarchical clustering analysis was used to identify gene modules and color to indicate modules, which is a cluster of densely interconnected genes in terms of co-expression. For genes that are not assigned to any of the modules, WGCNA places them in a grey module as not co-expressed. The module eigengene (ME) of a module is defined as the first principal component of the module and represents the overall expression level of the module. To identify modules that significantly associated with the traits of different etiologies, it was calculated the correlation of MEs (i.e., the first principal component of a module) [29] with clinical pathological features and identified the most significant associations. 

### 4.4. Functions and Pathways Interactome Analysis 

Significance Genes associated with ischemic pathological phenotype were analyzed using the web based Metascape, which integrates multiple authoritative data resources, such as GO, KEGG, UniProt and DrugBank, and executes pathway enrichment and biological process comprehensive annotation for each gene [30]. The gene enrichment *p*-values were calculated using the roast function of limma MethylMix. Only terms with *p* < 0.01, a minimum count of 3 and an enrichment factor > 1.5 were considered significant. 

### 4.5. Identification of Hub Genes Linked to ISCM

The module membership (MM) was defined as the correlation of gene expression profile with module eigengene (ME). The GS measure was defined as (the absolute value of) the correlation between gene and external traits. Hub genes were defined as a gene that in a module play important roles in the biological processes than other genes in the whole network, which were comprised as key interconnected nodes within a functionally network and played important roles in biological functions [31]. There are two methods, co-expression network and PPI network analysis, which have been employed to identify the real Hub genes among each significant module. Genes with the highest MM and highest GS in modules were processed as candidates for further functional research [32,33,34]. In this study, the criterial of screening hub genes were set up as GS > 0.2 and MM > 0.8 with a threshold of *p*-value < 0.05, and hub genes were identified in the most significantly module that correlated to certain clinical trait. In parallel, the protein–protein interaction (PPI) network of the module genes was built in the selected modules through STRING database. The significant module contained genes interaction between genes was defined as positive with a combined with the cutoff of >0.4 and connectivity degree of ≥8 through STRING database [35]. In the PPI network, filtered genes were defined as hub genes. The overlapped genes in both co-expression network and PPI network were regarded as “real” hub genes pickup for further analyses. Then, integration of protein–protein interaction (PPI) networks was visualized using Cytoscape software. The PPI network consists of 199 proteins (nodes) and 3240 directed edges, where the node size is stand for the degree value and the edge weight corresponds to the confidence of the predicted direction. There are 95 genes were screened out by setting the degree value above 8. These genes with higher degree of connectivity are in the core of PPI network. Genes that have been reported to link with pathological features were labelled with red color.

### 4.6. Significant Analysis for Signature Genes Expression 

Bead summary intensities were log2-transformed and quantile normalized for transcriptome analysis using the limma package and scripts in R/Bioconductor [36]. The Significance Analysis of Microarrays (SAM) was used to identify genes that associated with the ischemic cardiomyopathy when compared to health control group. Genes with Delta > 6.997 and a false discovery rate below 0.05 were considered as significant (Supplementary Table S4). The health group was used as the benchmark. The individual gene expression in each group was presented as means ± standard error of the mean (SEM) that represent cases distribution of group. The expression level comparison used the fold change ratio to quantitatively analyze. 

### 4.7. Immunohistochemistry and H&E Staining 

Briefly, standard immunohistochemistry (IHC) was performed on experimental rat heart using microwave in 0.1M citrate buffer (pH 6.0) as the antigen retrieval method. The target proteins antibodies (cTn, IFIT2, IFIT3, IFI44L, BCL2L1) were got from Abcam or Bioss. Following incubation, the reaction was visualized using Vectastain Elite ABC Kit with diaminobenzidine chromogen as a substrate and lightly counterstained with hematoxylin and mounted. Immunohistochemical results of stain slides were observed and evaluated by ImageJ software and IHC Profiler plugin [37]. The intensity of slide immunohistochemistry was scored automatically after the slides counting. The IHC scored values are defined as (1) Negative, (2) Low positive, (3) Positive and (4) High positive.

### 4.8. Quantitative Real-Time PCR (qPCR)

Total RNA was extracted from rat heart with TREzol (Invitrogen). Expression levels of target signature genes mRNA were analyzed with SYBR green-based real-time quantitative PCR assays (7500 FAST Real-Time PCR System, Applied Biosystems, Invitrogen; GoScript™ Reverse Transcription System, A5001, Promega; GoTaq^®^ qPCR Master Mix, A6001, Promega), with GAPDH as the internal control. The primers of the target genes used for qPCR assays are listed (Supplementary Table S7). The relative gene expression was determined by Delta CT Method. The significance of differences genes expression was determined between health and cardiomyopathies cases by using Student’s *t*-test with the Prism software (GraphPad Software, Inc. San Diego, CA, USA). A *p*-value < 0.05 was set up as significant difference standard (*, represents *p*-value < 0.05; **, represents 0.001 < *p*-value < 0.0001; ***, represents *p*-value < 0.0001). The standard of significance was set up as up-expression (Fold change > 1.0, *p* < 0.05) or down-expression (Fold change < 1.0, *p* < 0.05). 

### 4.9. Elisa Detection Target Proteins

The protein in the rat serum was diluted to 50 µg/mL with coating buffer (pH 9.6, composed of 0.015 M Na_2_CO_3_ and 0.035 M NaHCO_3_). Following that, the serum dilution (50 µL) was coated in Costar clear polystyrene high-protein-binding enzyme immunoassay (EIA) plates (Corning, Lowell, MA, USA). After overnight incubation at 2–6 °C, the dilution was discarded and the well was washed four times with 200 µL of wash buffer (pH = 7.4, phosphate-buffered saline containing 0.1% Tween 20), 10 min each time. After that, the plates were blocked with 200 µL wash buffer (containing 1% bovine serum albumin) each well. Incubate for 90 min at room temperature. Wash the plates twice with PBS-T. The 50 µL primary antibody (Rabbit Anti-IFIT2: bs-15528R, Bioss; Rabbit Anti-IFI44L: bs-15551R, Bioss; Rabbit Anti-IFIT3: A3924, ABclonal; Rabbit Anti-BCL2L1: A0209, ABclonal; Rabbit Anti-TNNT2: A1126, ABclonal.) dilution, diluted to 1:2000 times with 1% BSA/PBS, were conjugated with the rat sera. After overnight incubation at 2–6 °C, the well contents were recovered and washed four times with wash buffer for 10 min each time. Pipette 200 µL solution of the goat anti-rabbit IgG/horseradish peroxidase conjugate (HRP-labeled goat anti-rabbit IgG (H+L) (A0208, Beyotime, Shanghai, China), (diluted to 1:250 times with 1% BSA/PBS) into each of the wells and incubate for three hours at 37 °C. Remove the unbound conjugate anti-body by washing the plate three times with PBS-T. Fifty microliters of the substrate reagent, tetramethylbenzidine (TMB) (Substrate Solution for ELISA, P0209, Beyotime), was then added to each well. After 40 min incubation at room temperature, the reaction was stopped by the addition of 50 µL stop solution (Stop solution for TMB Substrate, P0215, Sulfuric acid free, 450 nm, Beyotime, Shanghai, China). Optical densities (OD) at 450 nm were determined using an ELISA plate reader (ELX 808 microplate reader; BioTek Instruments, Inc., Winooski, VT, USA). 

### 4.10. Animals Experimental Protocol

Hunan SJA Laboratory Animal Co. Ltd., People’s Republic of China (license No. SCXK (Xiang) 2019-0004) provided the adult male specific pathogen-free Sprague–Dawley (SD) rats (220–250 g). Before the experiment, rats were kept in a temperature-controlled room (24 °C, 50–60% relative humidity) with a 12-h light–dark cycle and acclimatized to the laboratory environment for 7 days by feeding them a standard diet and water. The Committee of Institutional Animal Care and Usage, Kunming Institute of Botany, Chinese Academy of Sciences reviewed and approved all experimental protocols (Permit No. KIB-R-018). Experiments were performed in the animal facility accordance to the research guide of Institutive Lab Research Animal Care & Usage. 

### 4.11. Rat Surgical Protocol and Cardiac Function Evaluation

The rats were divided into three groups at random: positive control (*n* = 12), left anterior descending (Lad) (*n* = 21), and sham (*n* = 22). The positive control group received metoprolol (2.0 mg/kg; IV; CAS: 56392-17-7, 98%, Aladdin) immediately after occlusion. The Lad and sham groups received the same volume of normal saline. Furthermore, to control for the effects of anesthesia and the operation process on cardiac function, the sham-operated group received anesthesia and a thoracotomy without Lad occlusion. Rats were sedated with 4% isoflurane in oxygen and kept sedated with 2% isoflurane.

Following induction, rats were used for left anterior descending (Lad) coronary artery ligation to induce myocardial infarction [18] and left coronary artery ligation according to previously described methodology [38]. A small (2 cm) skin cut was made over the left thoracotomy. The 4th intercostal space was exposed after the pectoral major and minor muscles were dissected and retracted. To open the pleural membrane and pericardium, a small hole was made at the 4th intercostal space with a mosquito clamp. The heart was smoothly and gently “popped out” through the hole with the clamp slightly open. The left auricle was gently retracted, exposing the left anterior descending coronary artery. Using a 6–0 silk suture, the left main descending coronary artery (LCA) was located, sutured, and ligated about 3 mm from its origin. When the anterior wall of the LV turned pale, the ligation was deemed successful. Following ligation, the heart was immediately returned to the intra-thoracic space, followed by manual air evacuation and muscle and skin closure. The mouse was then allowed to breathe room air and was monitored during the recovery period, which took about 1 min. During the recovery period, no artificial respiratory aid was required. The sham group underwent the same surgical procedure as the control group, with the exception that the Lad was not occluded.

### 4.12. Rat Cardiac Function and Heart Infarct Size Assessment

After a myocardial infarction, the size of the infarction and cardiac dysfunction were measured using echocardiography (Ultrasound echo-color Doppler system, VEVO3100, FUJIFILM Visual Sonics; Mindray M9 Echography, Mindray Co, Guangdong, China) and triphenyl-tetrazolium chloride (TTC) staining, respectively [39]. Briefly, TTC staining was used to determine the size of the myocardial infarct 24 h after the MI. Rats were killed at the end of the experiment via heart excision under deep anesthesia with 5% isoflurane. The heart was removed and divided into five equal slices. Histochemical staining with phosphotungstic acid hematoxylin was used to distinguish viable myocardium from infarcted myocardium. Stained slides were scanned, and the infarcted area and total heart area were marked manually using the program Pannoramic viewer (version 1.15.4). The average infarct size in each of five slices was calculated as a percentage of the entire transverse heart section.

### 4.13. Evaluation of Cardiomyocytes Apoptosis

The heart was cut into 5 µm slices by freezing microtome and then were fixed with acetone for 1 min. Subsequently, the sections were washed by PBS buffer for three times and were incubation in 5% Triton X-100 solution for 5 min at room temperature. After being washed three times, the sections were incubated with TUNEL reagents (One step TUNEL apoptosis assay kit, green fluorescence, Cat NO.1088, Beyotime) for one hour at 37 °C. Next, the sections were washed three times by PBS buffer to remove residual reagents. Finally, the antifade Mounting Medium with DAPI (Cat NO. P0131, Beyotime) was added in the slice and detected in the Fluorescence microscope. All procedures are executed strictly according to the protocol.

## 5. Conclusions

In summary, we integrated clinical patients’ tissue transcriptional expression data with an animal MI model to discover and validate the potential biomarkers for ischemic cardiomyopathy. These results expanded our understanding of potential novel key regulatory biomarkers or signature genes for ISCM. IFIT*2,* IFIT3 and IFI*44L* have been identified as potential clinical therapy targets.

## Data Availability

The microarray data generated or analyzed during this study were deposited in Gene Expression Omnibus (GEO; http://www.ncbi.nlm.nih.gov/projects/geo/; accession GSE57338, accessed on 1 January 2015), including original files and normalized data for next step analysis. Expression data were normalized, background-corrected and log2-transformed for parametric analysis.

## Supplementary Materials

The following are available online at https://www.mdpi.com/article/10.3390/ijms222313116/s1.
