# Supplementary material for: Identified Three Interferon Induced Proteins as Novel Biomarkers of Human Ischemic Cardiomyopathy
Source: Int J Mol Sci. 2021 Dec 4;22(23):13116. doi: 10.3390/ijms222313116 (PMC8657967; doi:10.3390/ijms222313116)
Supplement: Supplementary file 1 [file ijms-22-13116-s001.zip › ijms-1429438-supplementary figures-tables.pdf]

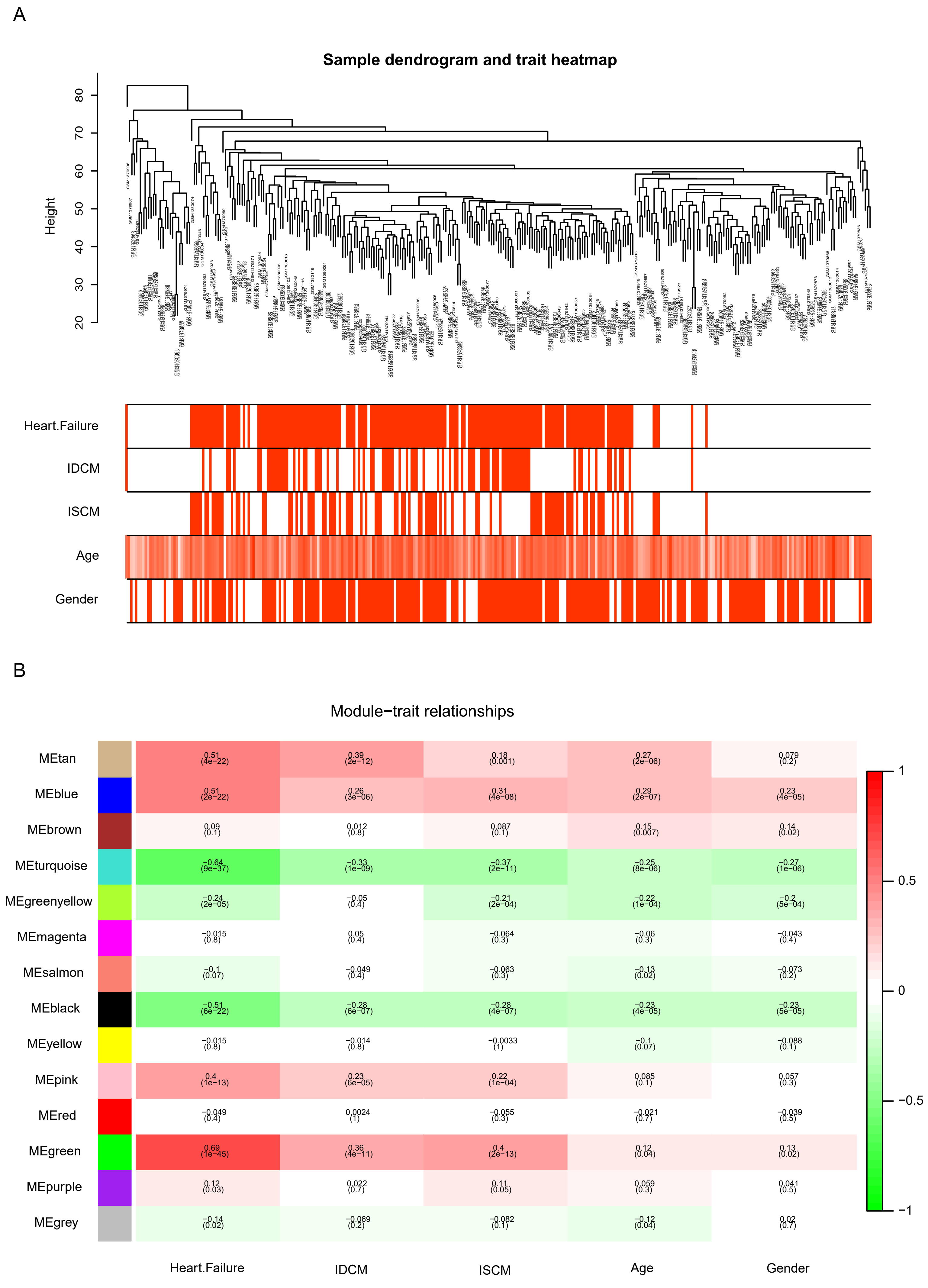
A
Sample dendrogram and trait heatmap
Height
80
70
60
50
40
30
20
Heart.Failure
IDCM
ISCM
Age
Gender
B
Module−trait relationships
MEtan
0.51 (4e−22)
0.39 (2e−12)
0.18 (0.001)
0.27 (2e−06)
0.079 (0.2)
MEblue
0.51 (2e−22)
0.26 (3e−06)
0.31 (4e−08)
0.29 (2e−07)
0.23 (4e−05)
MEbrown
0.09 (0.1)
0.012 (0.8)
0.087 (0.1)
0.15 (0.007)
0.14 (0.02)
MEturquoise
−0.64 (9e−37)
−0.33 (1e−09)
−0.37 (2e−11)
−0.25 (8e−06)
−0.27 (1e−06)
MEgreenyellow
−0.24 (2e−05)
−0.05 (0.4)
−0.21 (2e−04)
−0.22 (1e−04)
−0.2 (5e−04)
MEmagenta
−0.015 (0.8)
0.05 (0.4)
−0.064 (0.3)
−0.06 (0.3)
−0.043 (0.4)
MEsalmon
−0.1 (0.07)
−0.049 (0.4)
−0.063 (0.3)
−0.13 (0.02)
−0.073 (0.2)
MEblack
−0.51 (6e−22)
−0.28 (6e−07)
−0.28 (4e−07)
−0.23 (4e−05)
−0.23 (5e−05)
MEyellow
−0.015 (0.8)
−0.014 (0.8)
−0.0033 (1)
−0.1 (0.07)
−0.088 (0.1)
MEpink
0.4 (1e−13)
0.23 (6e−05)
0.22 (1e−04)
0.085 (0.1)
0.057 (0.3)
MEred
−0.049 (0.4)
0.0024 (1)
−0.055 (0.3)
−0.021 (0.7)
−0.039 (0.5)
MEgreen
0.69 (1e−45)
0.36 (4e−11)
0.4 (2e−13)
0.12 (0.04)
0.13 (0.02)
MEpurple
0.12 (0.03)
0.022 (0.7)
0.11 (0.05)
0.059 (0.3)
0.041 (0.5)
MEgrey
−0.14 (0.02)
−0.069 (0.2)
−0.082 (0.1)
−0.12 (0.04)
0.02 (0.7)
Heart.Failure
IDCM
ISCM
Age
Gender
1
0.5
0
−0.5
−1

Supplementary Figure S1. The sample dendrogram and trait heatmap. The distinct co-expression modules were identified that significantly related to different pathological features (A). Clustering dendrogram of samples based on their Euclidean distance. The clinical feature traits were heart failure, IDCM and ISCM, gender and age. IDCM: Idiopathic Di1ated Cardiomyopathy; ISCM: ischemic cardiomyopathy. The white color means a low value, red means a high value. There are three case groups were separated by performed hierarchical clustering, were heart failure, IDCM and ISCM, respectively. (B) The correlation of Module-clinical traits. Each row corresponds to a module; each column corresponds to a clinical trait feature. Each cell contains the test statistic value and its corresponding p value from the linear mixed-effects model. Network of eigengene represents the relationships among the modules and the histological traits. There are fourteen modules were detected through the dataset. Four modules were significantly positive correlated to ISCM pathological feature, including tan (t-value = 0.18, p-value = 0.001), blue (t-value = 0.31, p-value = 4e-08), pink (t-value = 0.22, p-value = 1e-04), green (t-value = 0.4, p-value = 2e-13). The green module was the most significant correlation to ischemic cardiomyopathy.

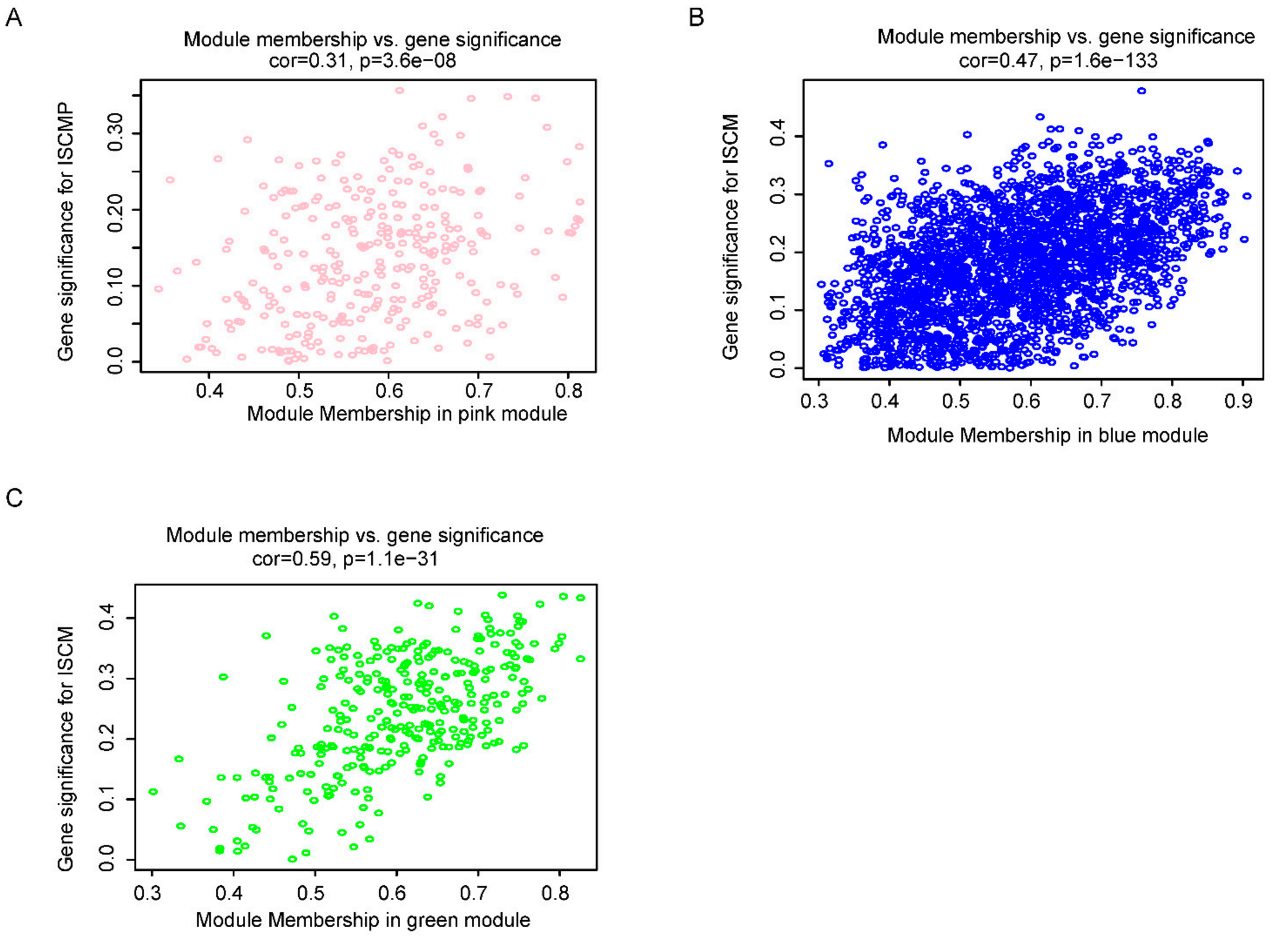

Supplementary Figure S2. The scatterplots of Gene Significance (GS) for histology vs. Module Membership (MM) in the significant modules (A~C). There is a highly significant correlation between GS and MM in this module, implying that the most important (central) elements of blue module also tend to be highly correlated with ISCM pathological trait. The green module (t-value = 0.59, p-value = 1.1e−31) has the highest correlation with ischemic cardiomyopathy status. It suggested that these gene contained in green module were significant associated with ischemic cardiomyopathy.

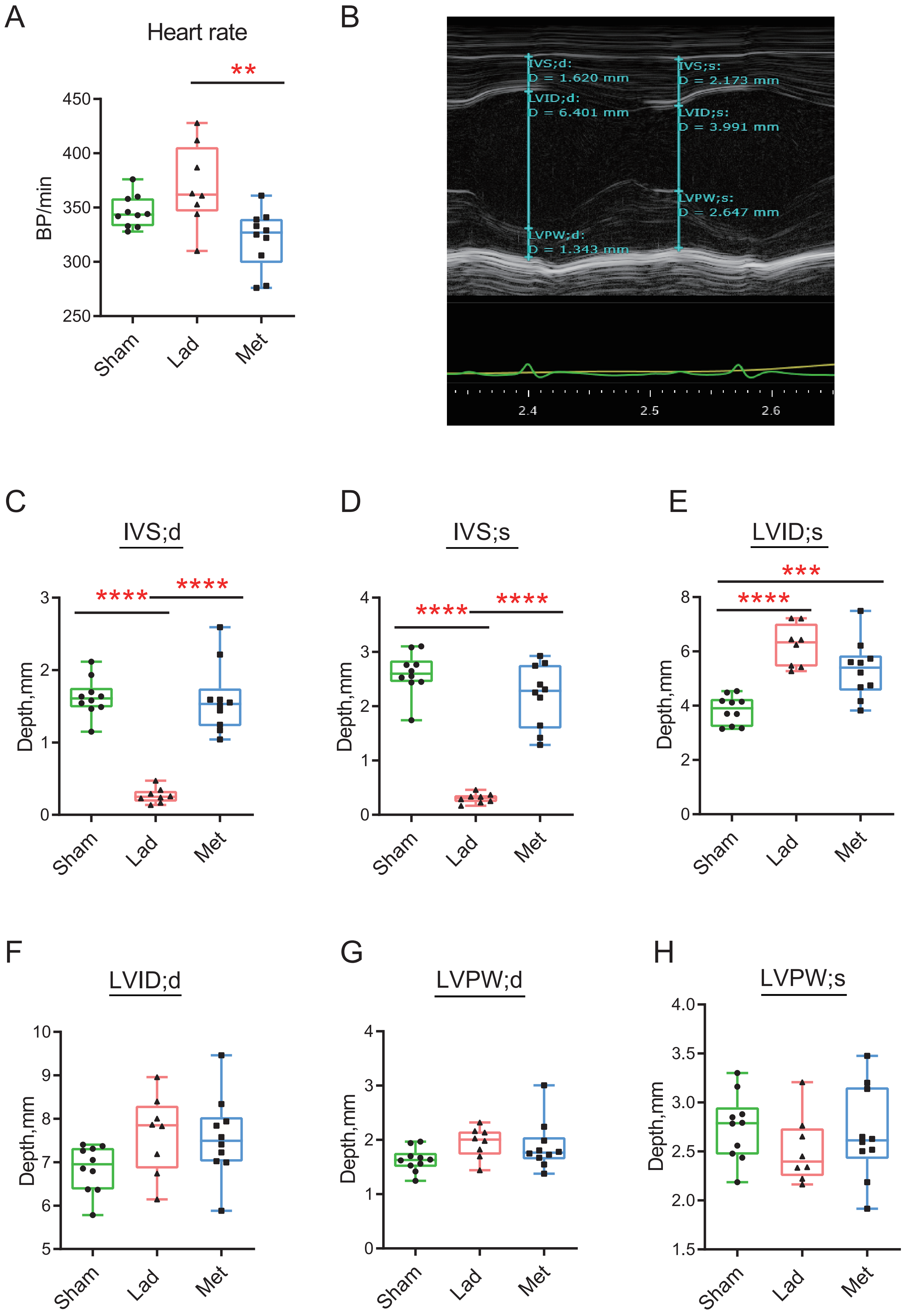

A
Heart rate
**
450
400
350
300
250
BP/min
Sham
Lad
Met
B
IVS;d:
D = 1.620 mm
LVID;d:
D = 6.401 mm
LVPW;d:
D = 1.343 mm
IVS;s:
D = 2.173 mm
LVID;s:
D = 3.991 mm
LVPW;s:
D = 2.647 mm
2.4
2.5
2.6
C
IVS;d
****
****
Depth,mm
D
IVS;s
****
****
Depth,mm
E
LVID;s
***
****
Depth,mm
F
LVID;d
Depth,mm
G
LVPW;d
Depth,mm
H
LVPW;s
Depth,mm
Sham
Lad
Met

Supplementary Figure S3. The heart rate and cardiac function after administration metoprolol for 3 days. The heart rate of rat in Sham group and LAD group were 346.2 ± 4.664 (n=10) and 369.8 ± 13.44 (n=8), respectively (A). Treatment with metoprolol decreased the rat's heart rate to 321.0 ± 8.595 (**P<0.01, n=10). Evaluation methods of cardiac function (B). LVEF: left ventricular ejection fraction; LVFS: left ventricular fractional shortening; IVSs, interventricular septal thickness in systole; LVIDs: left ventricular internal diameter in systole; LVPWs: LV posterior wall thickness in systole; IVSd: interventricular septal thickness in diastole; LVIDd: left ventricular internal dimension-diastole; LVPWd: LV posterior wall thickness in diastole (C)-(H). Results were expressed as the means±SEM and a dependent t-test would be used. **P<0.001 and ****P<0.0001. Sham group, n=10; Lad group, n=8; MET group, n=10.

Table. S1 The sample information about expression profile.

Sample ID	heart failure State	Normal	IDCM	ISCM	gender
GSM1380124	0	1	0	0	1
GSM1380123	0	1	0	0	0
GSM1380122	0	1	0	0	1
GSM1380027	0	1	0	0	1
GSM1380021	0	1	0	0	1
GSM1380015	0	1	0	0	0
GSM1380014	0	1	0	0	0
GSM1380013	0	1	0	0	0
GSM1380012	0	1	0	0	1
GSM1380011	0	1	0	0	1
GSM1380010	0	1	0	0	0
GSM1380008	0	1	0	0	0
GSM1379991	0	1	0	0	1
GSM1379990	0	1	0	0	1
GSM1379989	0	1	0	0	1
GSM1379988	0	1	0	0	1
GSM1379987	0	1	0	0	1
GSM1379986	0	1	0	0	1
GSM1379985	0	1	0	0	0
GSM1379984	0	1	0	0	0
GSM1379983	0	1	0	0	0
GSM1379982	0	1	0	0	0
GSM1379981	0	1	0	0	0
GSM1379980	0	1	0	0	1
GSM1379979	0	1	0	0	1
GSM1379978	0	1	0	0	1
GSM1379977	0	1	0	0	0
GSM1379976	0	1	0	0	0
GSM1379975	0	1	0	0	1
GSM1379974	0	1	0	0	0
GSM1379973	0	1	0	0	0
GSM1379972	0	1	0	0	0
GSM1379971	0	1	0	0	1
GSM1379970	0	1	0	0	1
GSM1379969	0	1	0	0	0
GSM1379968	0	1	0	0	1
GSM1379967	0	1	0	0	0
GSM1379966	0	1	0	0	0
GSM1379965	0	1	0	0	1
GSM1379962	0	1	0	0	0
GSM1379961	0	1	0	0	1
GSM1379927	0	1	0	0	0
GSM1379926	0	1	0	0	0
GSM1379925	0	1	0	0	1
GSM1379924	0	1	0	0	1
GSM1379923	0	1	0	0	1
GSM1379922	0	1	0	0	0
GSM1379921	0	1	0	0	0
GSM1379920	0	1	0	0	1
GSM1379919	0	1	0	0	1
GSM1379918	0	1	0	0	1

GSM1379915	0	1	0	0	0
GSM1379914	0	1	0	0	1
GSM1379913	0	1	0	0	0
GSM1379912	0	1	0	0	1
GSM1379911	0	1	0	0	0
GSM1379910	0	1	0	0	1
GSM1379909	0	1	0	0	0
GSM1379908	0	1	0	0	0
GSM1379907	0	1	0	0	0
GSM1379906	0	1	0	0	0
GSM1379905	0	1	0	0	0
GSM1379904	0	1	0	0	1
GSM1379903	0	1	0	0	1
GSM1379902	0	1	0	0	0
GSM1379901	0	1	0	0	1
GSM1379900	0	1	0	0	0
GSM1379899	0	1	0	0	1
GSM1379898	0	1	0	0	0
GSM1379897	0	1	0	0	0
GSM1379896	0	1	0	0	0
GSM1379895	0	1	0	0	0
GSM1379894	0	1	0	0	0
GSM1379893	0	1	0	0	0
GSM1379892	0	1	0	0	0
GSM1379891	0	1	0	0	1
GSM1379890	0	1	0	0	1
GSM1379889	0	1	0	0	1
GSM1379888	0	1	0	0	1
GSM1379887	0	1	0	0	1
GSM1379886	0	1	0	0	0
GSM1379885	0	1	0	0	1
GSM1379884	0	1	0	0	1
GSM1379883	0	1	0	0	1
GSM1379882	0	1	0	0	1
GSM1379881	0	1	0	0	1
GSM1379880	0	1	0	0	1
GSM1379878	0	1	0	0	1
GSM1379877	0	1	0	0	0
GSM1379876	0	1	0	0	0
GSM1379875	0	1	0	0	0
GSM1379874	0	1	0	0	1
GSM1379873	0	1	0	0	1
GSM1379872	0	1	0	0	1
GSM1379871	0	1	0	0	0
GSM1379870	0	1	0	0	1
GSM1379869	0	1	0	0	0
GSM1379868	0	1	0	0	1
GSM1379867	0	1	0	0	0
GSM1379866	0	1	0	0	0
GSM1379865	0	1	0	0	1
GSM1379864	0	1	0	0	1
GSM1379863	0	1	0	0	0
GSM1379862	0	1	0	0	1
GSM1379861	0	1	0	0	1

GSM1379860	0	1	0	0	1
GSM1379859	0	1	0	0	1
GSM1379858	0	1	0	0	1
GSM1379857	0	1	0	0	1
GSM1379856	0	1	0	0	1
GSM1379855	0	1	0	0	0
GSM1379854	0	1	0	0	1
GSM1379853	0	1	0	0	0
GSM1379852	0	1	0	0	0
GSM1379851	0	1	0	0	1
GSM1379850	0	1	0	0	1
GSM1379849	0	1	0	0	0
GSM1379848	0	1	0	0	1
GSM1379847	0	1	0	0	0
GSM1379846	0	1	0	0	1
GSM1379845	0	1	0	0	1
GSM1379844	0	1	0	0	1
GSM1379843	0	1	0	0	1
GSM1379842	0	1	0	0	0
GSM1379841	0	1	0	0	1
GSM1379840	0	1	0	0	1
GSM1379839	0	1	0	0	1
GSM1379838	0	1	0	0	0
GSM1379837	0	1	0	0	1
GSM1379836	0	1	0	0	0
GSM1379835	0	1	0	0	0
GSM1379834	0	1	0	0	0
GSM1379833	0	1	0	0	1
GSM1379832	0	1	0	0	0
GSM1379831	0	1	0	0	0
GSM1379830	0	1	0	0	0
GSM1380120	1	0	0	1	1
GSM1380119	1	0	0	1	1
GSM1380117	1	0	0	1	1
GSM1380116	1	0	0	1	1
GSM1380115	1	0	0	1	1
GSM1380114	1	0	0	1	1
GSM1380113	1	0	0	1	1
GSM1380112	1	0	0	1	1
GSM1380111	1	0	0	1	1
GSM1380110	1	0	0	1	1
GSM1380109	1	0	0	1	1
GSM1380108	1	0	0	1	1
GSM1380107	1	0	0	1	0
GSM1380106	1	0	0	1	0
GSM1380105	1	0	0	1	1
GSM1380104	1	0	0	1	1
GSM1380101	1	0	0	1	0
GSM1380100	1	0	0	1	1
GSM1380097	1	0	0	1	1
GSM1380093	1	0	0	1	1
GSM1380092	1	0	0	1	0
GSM1380091	1	0	0	1	1
GSM1380090	1	0	0	1	1

GSM1380089	1	0	0	1	1
GSM1380087	1	0	0	1	1
GSM1380086	1	0	0	1	1
GSM1380081	1	0	0	1	0
GSM1380079	1	0	0	1	1
GSM1380076	1	0	0	1	1
GSM1380074	1	0	0	1	0
GSM1380071	1	0	0	1	1
GSM1380070	1	0	0	1	0
GSM1380069	1	0	0	1	1
GSM1380067	1	0	0	1	1
GSM1380066	1	0	0	1	1
GSM1380065	1	0	0	1	1
GSM1380064	1	0	0	1	1
GSM1380063	1	0	0	1	1
GSM1380061	1	0	0	1	1
GSM1380060	1	0	0	1	1
GSM1380057	1	0	0	1	1
GSM1380055	1	0	0	1	0
GSM1380054	1	0	0	1	1
GSM1380052	1	0	0	1	1
GSM1380051	1	0	0	1	1
GSM1380049	1	0	0	1	1
GSM1380047	1	0	0	1	1
GSM1380045	1	0	0	1	1
GSM1380044	1	0	0	1	1
GSM1380043	1	0	0	1	1
GSM1380042	1	0	0	1	0
GSM1380041	1	0	0	1	1
GSM1380040	1	0	0	1	1
GSM1380030	1	0	0	1	1
GSM1380024	1	0	0	1	1
GSM1380019	1	0	0	1	1
GSM1380018	1	0	0	1	1
GSM1380007	1	0	0	1	1
GSM1380005	1	0	0	1	1
GSM1380004	1	0	0	1	1
GSM1380002	1	0	0	1	1
GSM1380001	1	0	0	1	1
GSM1380000	1	0	0	1	1
GSM1379999	1	0	0	1	1
GSM1379998	1	0	0	1	1
GSM1379997	1	0	0	1	1
GSM1379992	1	0	0	1	1
GSM1379964	1	0	0	1	1
GSM1379960	1	0	0	1	1
GSM1379959	1	0	0	1	1
GSM1379958	1	0	0	1	1
GSM1379953	1	0	0	1	1
GSM1379952	1	0	0	1	1
GSM1379951	1	0	0	1	1
GSM1379949	1	0	0	1	0
GSM1379948	1	0	0	1	1
GSM1379946	1	0	0	1	0

GSM1379945	1	0	0	1	1
GSM1379943	1	0	0	1	1
GSM1379938	1	0	0	1	1
GSM1379933	1	0	0	1	1
GSM1379930	1	0	0	1	1
GSM1379928	1	0	0	1	1
GSM1379829	1	0	0	1	1
GSM1379828	1	0	0	1	1
GSM1379826	1	0	0	1	1
GSM1379825	1	0	0	1	0
GSM1379824	1	0	0	1	1
GSM1379823	1	0	0	1	1
GSM1379822	1	0	0	1	1
GSM1379821	1	0	0	1	1
GSM1379819	1	0	0	1	0
GSM1379815	1	0	0	1	1
GSM1379814	1	0	0	1	1
GSM1379813	1	0	0	1	0
GSM1380121	1	0	1	0	0
GSM1380118	1	0	1	0	1
GSM1380103	1	0	1	0	0
GSM1380102	1	0	1	0	0
GSM1380099	1	0	1	0	1
GSM1380098	1	0	1	0	0
GSM1380096	1	0	1	0	0
GSM1380095	1	0	1	0	0
GSM1380094	1	0	1	0	0
GSM1380088	1	0	1	0	1
GSM1380085	1	0	1	0	1
GSM1380084	1	0	1	0	1
GSM1380083	1	0	1	0	1
GSM1380082	1	0	1	0	1
GSM1380080	1	0	1	0	1
GSM1380078	1	0	1	0	1
GSM1380077	1	0	1	0	1
GSM1380075	1	0	1	0	1
GSM1380073	1	0	1	0	1
GSM1380072	1	0	1	0	0
GSM1380068	1	0	1	0	0
GSM1380062	1	0	1	0	1
GSM1380059	1	0	1	0	1
GSM1380058	1	0	1	0	1
GSM1380056	1	0	1	0	1
GSM1380053	1	0	1	0	1
GSM1380050	1	0	1	0	1
GSM1380048	1	0	1	0	1
GSM1380046	1	0	1	0	1
GSM1380039	1	0	1	0	1
GSM1380038	1	0	1	0	0
GSM1380037	1	0	1	0	1
GSM1380036	1	0	1	0	1
GSM1380035	1	0	1	0	1
GSM1380034	1	0	1	0	0
GSM1380033	1	0	1	0	0

GSM1380032	1	0	1	0	1
GSM1380031	1	0	1	0	1
GSM1380029	1	0	1	0	1
GSM1380028	1	0	1	0	1
GSM1380026	1	0	1	0	1
GSM1380025	1	0	1	0	1
GSM1380023	1	0	1	0	0
GSM1380022	1	0	1	0	1
GSM1380020	1	0	1	0	1
GSM1380017	1	0	1	0	1
GSM1380016	1	0	1	0	1
GSM1380009	1	0	1	0	1
GSM1380006	1	0	1	0	1
GSM1380003	1	0	1	0	1
GSM1379996	1	0	1	0	0
GSM1379995	1	0	1	0	0
GSM1379994	1	0	1	0	1
GSM1379993	1	0	1	0	0
GSM1379963	1	0	1	0	1
GSM1379957	1	0	1	0	1
GSM1379956	1	0	1	0	1
GSM1379955	1	0	1	0	1
GSM1379954	1	0	1	0	1
GSM1379950	1	0	1	0	1
GSM1379947	1	0	1	0	1
GSM1379944	1	0	1	0	1
GSM1379942	1	0	1	0	0
GSM1379941	1	0	1	0	1
GSM1379940	1	0	1	0	1
GSM1379939	1	0	1	0	1
GSM1379937	1	0	1	0	1
GSM1379936	1	0	1	0	1
GSM1379935	1	0	1	0	1
GSM1379934	1	0	1	0	0
GSM1379932	1	0	1	0	1
GSM1379931	1	0	1	0	0
GSM1379929	1	0	1	0	1
GSM1379917	1	0	1	0	1
GSM1379916	1	0	1	0	1
GSM1379879	1	0	1	0	1
GSM1379827	1	0	1	0	1
GSM1379820	1	0	1	0	1
GSM1379818	1	0	1	0	1
GSM1379817	1	0	1	0	1
GSM1379816	1	0	1	0	1
GSM1379812	1	0	1	0	1

Table.S2 The signficance genes related to ISCM.

GeneSymbol	moduleColor	GS.ISCMP	p.GS.ISCMP	MM.green	p.MM.green
OGN	green	0.4380	5.21E-16	0.7285	1.08E-52
ISLR	green	0.4356	7.86E-16	0.8034	1.49E-71
BTN3A1	green	0.4333	1.15E-15	0.8244	2.27E-78
IL1RL1	green	(0.4242)	5.14E-15	(0.6249)	4.37E-35
LUM	green	0.4226	6.64E-15	0.7747	1.77E-63
KCNN3	green	0.4199	1.03E-14	0.6388	4.61E-37
HTRA1	green	0.4109	4.24E-14	0.6743	1.37E-42
NTM	green	0.4048	1.08E-13	0.7077	1.56E-48
IFI44L	green	0.4033	1.35E-13	0.7473	8.54E-57
SSPN	green	0.4027	1.50E-13	0.5222	3.75E-23
CRISPLD1	green	0.3972	3.40E-13	0.7111	3.40E-49
SFRP4	green	0.3948	4.85E-13	0.7496	2.50E-57
COL14A1	green	0.3940	5.45E-13	0.7533	3.57E-58
ASPN	green	0.3862	1.68E-12	0.7479	6.06E-57
TRIM22	green	0.3828	2.72E-12	0.7132	1.33E-49
SPATA18	green	0.3824	2.87E-12	0.5327	3.39E-24
LCN6	green	(0.3810)	3.50E-12	(0.6715)	3.94E-42
C15orf59	green	(0.3801)	3.94E-12	(0.6011)	6.22E-32
FRZB	green	0.3751	7.89E-12	0.7260	3.62E-52
TIMP2	green	0.3749	8.16E-12	0.7386	7.52E-55
LRRC17	green	0.3736	9.78E-12	0.7147	6.76E-50
IL18R1	green	(0.3706)	1.48E-11	(0.4391)	4.31E-16
MFAP4	green	0.3704	1.50E-11	0.6990	6.50E-47
UBA7	green	0.3693	1.76E-11	0.8015	5.59E-71
XAF1	green	0.3658	2.79E-11	0.6981	9.55E-47
NRK	green	0.3657	2.81E-11	0.7020	1.82E-47
PCNT	green	(0.3617)	4.82E-11	(0.5718)	2.13E-28
SNCAIP	green	0.3609	5.35E-11	0.6178	4.09E-34
ACKR4	green	0.3595	6.40E-11	0.7451	2.64E-56
SLCO4A1	green	(0.3583)	7.47E-11	(0.6356)	1.34E-36
NT5E	green	0.3579	7.84E-11	0.7980	6.26E-70
PDE5A	green	0.3575	8.30E-11	0.7678	1.08E-61
NREP	green	0.3573	8.52E-11	0.6116	2.70E-33
C1QTNF2	green	0.3562	9.82E-11	0.7194	7.82E-51
HAAO	green	0.3541	1.28E-10	0.6320	4.45E-36
IFIT3	green	0.3533	1.43E-10	0.7495	2.71E-57
UBTD1	green	(0.3518)	1.73E-10	(0.5379)	1.01E-24
ACE	green	0.3515	1.78E-10	0.5748	9.58E-29
NR2F6	green	(0.3510)	1.91E-10	(0.5926)	7.17E-31
CCNG2	green	0.3507	1.98E-10	0.5169	1.21E-22
ST6GALNAC3	green	(0.3494)	2.33E-10	(0.6068)	1.16E-32
QTRT1	green	(0.3491)	2.41E-10	(0.6009)	6.53E-32
CTSK	green	0.3490	2.44E-10	0.7929	1.86E-68
WNK3	green	(0.3468)	3.21E-10	(0.5278)	1.06E-23
PRDM1	green	0.3463	3.42E-10	0.6503	8.93E-39
MARK3	green	(0.3458)	3.64E-10	(0.6463)	3.66E-38
ADAM22	green	0.3453	3.88E-10	0.5000	4.47E-21
FGF14	green	0.3453	3.90E-10	0.6252	4.00E-35
BOC	green	0.3451	4.00E-10	0.7006	3.27E-47
PHYHD1	green	(0.3444)	4.34E-10	(0.5872)	3.26E-30
PLEKHH2	green	0.3436	4.77E-10	0.6615	1.67E-40
ANTXR1	green	0.3429	5.23E-10	0.7345	5.68E-54
MXRA5	green	0.3412	6.40E-10	0.7406	2.74E-55
LCN10	green	(0.3392)	8.23E-10	(0.6419)	1.65E-37
BCL2L1	green	(0.3379)	9.57E-10	(0.5511)	4.15E-26

MAVS	green	(0.3357)	1.26E-09	(0.5754)	8.18E-29
EIF2AK2	green	0.3356	1.27E-09	0.5345	2.26E-24
SYTL2	green	0.3353	1.31E-09	0.7444	3.96E-56
PLA2R1	green	0.3351	1.34E-09	0.6248	4.44E-35
ZFP90	green	0.3346	1.42E-09	0.6382	5.65E-37
FAM102B	green	0.3325	1.84E-09	0.7588	1.70E-59
SAMD9L	green	0.3322	1.90E-09	0.8244	2.30E-78
ST6GALNAC3	green	(0.3314)	2.10E-09	(0.5929)	6.59E-31
HIP1R	green	(0.3313)	2.11E-09	(0.5186)	8.20E-23
DDX60	green	0.3306	2.29E-09	0.7622	2.66E-60
MX1	green	0.3302	2.42E-09	0.6707	5.35E-42
DIO2	green	0.3291	2.72E-09	0.5666	8.27E-28
EXT1	green	0.3278	3.20E-09	0.6268	2.39E-35
LPAR4	green	0.3277	3.22E-09	0.6817	7.64E-44
MYOF	green	0.3262	3.83E-09	0.6119	2.49E-33
SESN3	green	0.3246	4.62E-09	0.6878	6.82E-45
CRHBP	green	0.3237	5.14E-09	0.5538	2.15E-26
TMTC1	green	(0.3231)	5.44E-09	(0.6093)	5.39E-33
IFIT2	green	0.3223	6.00E-09	0.7380	1.03E-54
IFI44	green	0.3206	7.29E-09	0.6942	4.98E-46
RSAD2	green	0.3206	7.31E-09	0.6083	7.26E-33
TLR3	green	0.3190	8.70E-09	0.7314	2.65E-53
SULF1	green	0.3181	9.61E-09	0.7453	2.39E-56
DPYSL3	green	0.3166	1.15E-08	0.7446	3.47E-56
DDX58	green	0.3153	1.32E-08	0.7140	9.27E-50
MTSS1L	green	(0.3142)	1.49E-08	(0.5337)	2.68E-24
PRDX6	green	(0.3139)	1.53E-08	(0.6040)	2.62E-32
STAMBPL1	green	0.3127	1.76E-08	0.6952	3.16E-46
CXCL12	green	0.3117	1.97E-08	0.7089	8.88E-49
TRERF1	green	0.3109	2.15E-08	0.6444	6.87E-38
FRMD4B	green	0.3094	2.53E-08	0.6930	7.91E-46
EPHA3	green	0.3088	2.69E-08	0.6781	3.13E-43
IL34	green	0.3081	2.89E-08	0.6732	2.09E-42
ESRRA	green	(0.3078)	2.99E-08	(0.6088)	6.32E-33
PPP3CC	green	(0.3076)	3.07E-08	(0.5743)	1.11E-28
MAGED2	green	0.3068	3.33E-08	0.5534	2.40E-26
PI16	green	0.3059	3.68E-08	0.6301	8.28E-36
PRICKLE3	green	(0.3040)	4.54E-08	(0.5297)	6.78E-24
LSP1	green	0.3030	5.00E-08	0.6588	4.40E-40
CX3CL1	green	0.3023	5.39E-08	0.3864	1.62E-12
MYH10	green	0.3022	5.47E-08	0.6582	5.46E-40
OAS1	green	0.3014	5.93E-08	0.7410	2.26E-55
TPPP3	green	0.3007	6.42E-08	0.5634	1.92E-27
POPDC2	green	(0.2992)	7.47E-08	(0.5095)	6.01E-22
DPT	green	0.2984	8.18E-08	0.7077	1.52E-48
TET1	green	0.2971	9.37E-08	0.5400	6.20E-25
NFIC	green	(0.2964)	9.99E-08	(0.5812)	1.72E-29
IRX4	green	(0.2958)	1.07E-07	(0.6290)	1.15E-35
OMD	green	0.2953	1.12E-07	0.5855	5.27E-30
CD83	green	0.2951	1.14E-07	0.4604	1.02E-17
LIMA1	green	0.2946	1.21E-07	0.7550	1.37E-58
PLXNB1	green	(0.2942)	1.25E-07	(0.6122)	2.24E-33
ST7-OT3	green	(0.2941)	1.27E-07	(0.5629)	2.17E-27
SNTB1	green	0.2921	1.55E-07	0.6862	1.26E-44
IFI6	green	0.2917	1.62E-07	0.6388	4.56E-37
ENPP2	green	0.2911	1.72E-07	0.6272	2.06E-35
NR3C1	green	0.2911	1.73E-07	0.6260	3.05E-35
STARD5	green	0.2871	2.57E-07	0.5866	3.87E-30
LIPH	green	0.2862	2.82E-07	0.5062	1.21E-21

PRMT2	green	0.2858	2.93E-07	0.6365	1.01E-36
OAS2	green	0.2842	3.43E-07	0.6882	5.81E-45
JMJD1C	green	0.2837	3.62E-07	0.5862	4.26E-30
AQP3	green	(0.2829)	3.92E-07	(0.5510)	4.26E-26
GVINP1	green	0.2826	4.04E-07	0.6282	1.52E-35
HLA-DPB1	green	0.2822	4.18E-07	0.7603	7.46E-60
EPB41L2	green	0.2817	4.41E-07	0.5938	5.09E-31
PLCB1	green	0.2813	4.60E-07	0.5759	7.22E-29
BTN3A2	green	0.2805	4.97E-07	0.6511	6.85E-39
CDC42EP4	green	(0.2781)	6.23E-07	(0.5545)	1.79E-26
SLFN13	green	0.2754	8.12E-07	0.6354	1.43E-36
GNA14	green	0.2754	8.13E-07	0.7530	4.08E-58
ALDH1A2	green	0.2739	9.35E-07	0.5880	2.60E-30
MX2	green	0.2732	9.96E-07	0.7318	2.13E-53
COL15A1	green	0.2730	1.02E-06	0.6907	2.11E-45
HLA-DPB1	green	0.2720	1.11E-06	0.7179	1.57E-50
INMT	green	0.2694	1.43E-06	0.6136	1.49E-33
TRIL	green	0.2691	1.46E-06	0.6082	7.45E-33
TCF4	green	0.2682	1.60E-06	0.7092	7.92E-49
HLA-DPB1	green	0.2669	1.80E-06	0.7769	4.75E-64
OAS3	green	0.2665	1.87E-06	0.6604	2.51E-40
SLC7A6OS	green	(0.2620)	2.82E-06	(0.6424)	1.37E-37
SLC29A2	green	(0.2609)	3.10E-06	(0.6705)	5.89E-42
KCNJ11	green	(0.2600)	3.36E-06	(0.5921)	8.34E-31
ADGRA2	green	0.2599	3.41E-06	0.7062	2.93E-48
HLA-DOA	green	0.2598	3.43E-06	0.5928	6.70E-31
PDGFD	green	0.2592	3.61E-06	0.5360	1.58E-24
ANXA4	green	0.2586	3.84E-06	0.6905	2.24E-45
CCL5	green	0.2581	3.98E-06	0.6746	1.24E-42
HLA-E	green	0.2580	4.01E-06	0.7535	3.17E-58
HLA-E	green	0.2580	4.01E-06	0.7535	3.17E-58
TRIM6-TRIM34	green	0.2579	4.07E-06	0.5941	4.73E-31
CX3CR1	green	0.2572	4.33E-06	0.6527	3.91E-39
BTN3A3	green	0.2572	4.34E-06	0.7223	2.03E-51
ITGBL1	green	0.2557	4.93E-06	0.6073	9.75E-33
HLA-DOA	green	0.2529	6.34E-06	0.6539	2.52E-39
STON2	green	0.2522	6.73E-06	0.6165	6.01E-34
GBP7	green	0.2521	6.79E-06	0.4704	1.59E-18
DZIP1	green	0.2519	6.91E-06	0.6229	8.13E-35
KLRK1	green	0.2503	7.88E-06	0.6289	1.22E-35
CFAP77	green	(0.2501)	8.01E-06	(0.5454)	1.68E-25
GBP4	green	0.2493	8.63E-06	0.6415	1.86E-37
RNASET2	green	0.2486	9.12E-06	0.6705	5.82E-42
HLA-E	green	0.2476	9.94E-06	0.7460	1.64E-56
HMCN1	green	0.2476	9.99E-06	0.7066	2.50E-48
HLA-DQA1	green	0.2475	1.00E-05	0.6195	2.37E-34
HLA-DQA1	green	0.2475	1.00E-05	0.6195	2.37E-34
ACKR4	green	0.2474	1.02E-05	0.5208	5.13E-23
BCL2L13	green	(0.2458)	1.16E-05	(0.6576)	6.87E-40
ARHGAP28	green	0.2450	1.24E-05	0.5765	6.08E-29
CYP4B1	green	(0.2425)	1.53E-05	(0.5998)	8.99E-32
CD3G	green	0.2388	2.07E-05	0.5294	7.32E-24
BICC1	green	0.2348	2.88E-05	0.6805	1.24E-43
APLNR	green	0.2335	3.20E-05	0.5675	6.52E-28
FKBP5	green	(0.2317)	3.70E-05	(0.5375)	1.11E-24
PARP8	green	0.2311	3.86E-05	0.6814	8.55E-44
SAMD9	green	0.2306	4.02E-05	0.6927	9.15E-46
FCER1A	green	0.2301	4.18E-05	0.5867	3.74E-30
CD74	green	0.2300	4.22E-05	0.7248	6.33E-52

HLA-DQA2	green	0.2296	4.37E-05	0.5868	3.70E-30
OPA3	green	(0.2291)	4.52E-05	(0.5301)	6.28E-24
GPR174	green	0.2267	5.47E-05	0.6517	5.45E-39
HLA-F	green	0.2264	5.59E-05	0.7089	8.82E-49
IFIH1	green	0.2252	6.15E-05	0.6335	2.67E-36
ZNF423	green	0.2239	6.81E-05	0.6368	9.00E-37
HLA-DQA1	green	0.2236	6.95E-05	0.4581	1.52E-17
P2RX7	green	0.2222	7.75E-05	0.6395	3.69E-37
CD163L1	green	0.2222	7.77E-05	0.6150	9.69E-34
RCE1	green	0.2199	9.18E-05	0.6039	2.68E-32
HLA-DQA2	green	0.2195	9.53E-05	0.5804	2.12E-29
MEOX2	green	0.2194	9.60E-05	0.5813	1.69E-29
MARCKS	green	0.2191	9.77E-05	0.6925	9.89E-46
CA5B	green	0.2183	0.000104223	0.5929	6.50E-31
SAMD3	green	0.2168	0.000116087	0.5141	2.21E-22
FMOD	green	0.2165	0.000118876	0.6262	2.86E-35
ITGAL	green	0.2156	0.00012697	0.6683	1.31E-41
C1orf54	green	0.2153	0.000129775	0.5274	1.16E-23
PLCG2	green	0.2151	0.000132332	0.5970	2.05E-31
HLA-F	green	0.2141	0.000142397	0.7107	4.13E-49
APOBEC3G	green	0.2127	0.000157456	0.6103	4.03E-33
HLA-J	green	0.2125	0.000159168	0.6538	2.67E-39
EML4	green	0.2120	0.000165395	0.6815	8.49E-44
RGS4	green	0.2120	0.00016586	0.6403	2.82E-37
TIMM44	green	(0.2109)	0.0001798	(0.5390)	7.78E-25
DHX58	green	0.2090	0.000205156	0.6142	1.22E-33
FAM111A	green	0.2077	0.000226174	0.6503	9.04E-39
PARP15	green	0.2063	0.000248472	0.5716	2.22E-28
DDX60L	green	0.2063	0.000248707	0.5462	1.40E-25
HLA-F	green	0.2058	0.000258466	0.7035	9.46E-48
COL12A1	green	0.2032	0.000310458	0.6714	4.22E-42
HLA-DRA	green	0.2028	0.000319011	0.6894	3.51E-45
HLA-J	green	0.2018	0.000342745	0.6226	8.86E-35
TMEM255A	green	0.2016	0.000347438	0.4453	1.49E-16
HLA-DPA1	green	0.2014	0.000350376	0.5909	1.16E-30
SLC6A10P	green	(0.2009)	0.000362919	(0.5657)	1.05E-27
CYP27A1	green	0.1998	0.00039214	0.6255	3.58E-35
P2RY13	green	0.1990	0.000414331	0.5920	8.38E-31
B2M	green	0.1968	0.000480254	0.6502	9.45E-39
HLA-DRA	green	0.1967	0.000484192	0.6870	9.31E-45
HLA-DMA	green	0.1954	0.00052971	0.7243	7.92E-52
HLA-DMA	green	0.1954	0.00052971	0.7243	7.92E-52
HLA-G	green	0.1948	0.000549603	0.6448	6.13E-38
HLA-G	green	0.1948	0.000549603	0.6448	6.13E-38
HLA-DMA	green	0.1932	0.000612899	0.7188	1.03E-50
GBP3	green	0.1925	0.000641564	0.6776	3.86E-43
VCAM1	green	0.1908	0.000719932	0.5065	1.14E-21
HLA-DRA	green	0.1906	0.000728723	0.6858	1.52E-44
STAT2	green	0.1889	0.000814424	0.6081	7.74E-33
HLA-DMB	green	0.1888	0.000818203	0.7545	1.78E-58
USP2	green	(0.1888)	0.000819133	(0.5190)	7.57E-23
CASP1	green	0.1883	0.000846183	0.7087	9.98E-49
VCL	green	(0.1870)	0.000923145	(0.5608)	3.68E-27
HLA-A	green	0.1866	0.000947453	0.6561	1.16E-39
TARP	green	0.1864	0.000954462	0.5211	4.77E-23
CXCL10	green	0.1864	0.000957099	0.5015	3.26E-21
HLA-H	green	0.1864	0.00095796	0.6755	8.70E-43
PARP14	green	0.1863	0.000965516	0.6629	9.77E-41
MOXD1	green	0.1860	0.000980949	0.5653	1.18E-27

CD1C	green	0.1853	0.001029833	0.5055	1.42E-21
SCN10A	green	(0.1848)	0.001060531	(0.4787)	3.20E-19
HLA-DPA1	green	0.1846	0.001072767	0.5657	1.06E-27
HLA-DPA1	green	0.1846	0.001072767	0.5657	1.06E-27
CXCL9	green	0.1842	0.001099756	0.5121	3.47E-22
PSMB9	green	0.1842	0.001103684	0.6418	1.68E-37
PSMB9	green	0.1842	0.001103684	0.6418	1.68E-37
PSMB9	green	0.1842	0.001103684	0.6418	1.68E-37
CLEC4A	green	0.1824	0.001238039	0.5467	1.23E-25
HLA-DMB	green	0.1822	0.001252395	0.7458	1.90E-56
APOL6	green	0.1814	0.001313448	0.6103	4.04E-33
IL2RB	green	0.1805	0.001392706	0.5378	1.03E-24
LURAP1L	green	0.1798	0.00144946	0.5802	2.25E-29
RECK	green	0.1792	0.00151077	0.5549	1.65E-26
EPSTI1	green	0.1787	0.001558758	0.5882	2.49E-30
RTP4	green	0.1765	0.001777612	0.4746	7.10E-19
MTMR9LP	green	0.1761	0.001819381	0.4813	1.93E-19
HCP5	green	0.1757	0.001867492	0.6032	3.35E-32
APOBEC3D	green	0.1741	0.002057955	0.5063	1.20E-21
LRMP	green	0.1688	0.002828896	0.6313	5.58E-36
NRN1	green	0.1684	0.002889435	0.5547	1.73E-26
USP18	green	0.1665	0.003220969	0.3319	1.96E-09
HLA-A	green	0.1615	0.004302434	0.6287	1.30E-35
PLXDC2	green	0.1603	0.004607178	0.5928	6.73E-31
PYHIN1	green	0.1590	0.004941473	0.5041	1.91E-21
HLA-B	green	0.1589	0.004980097	0.6632	8.99E-41
STAT1	green	0.1581	0.005209595	0.6280	1.59E-35
NLRC5	green	0.1558	0.005907644	0.5449	1.91E-25
ARRB1	green	0.1545	0.006331276	0.5582	7.11E-27
RGL1	green	0.1531	0.00684546	0.5852	5.68E-30
HLA-DRB4	green	0.1521	0.007201436	0.5592	5.58E-27
HLA-DRB3	green	0.1520	0.007239531	0.5384	8.94E-25
LPXN	green	0.1469	0.009490672	0.5773	4.90E-29
GBP5	green	0.1459	0.010007854	0.5660	9.81E-28
PLXNC1	green	0.1450	0.01045412	0.6265	2.63E-35
KCNA3	green	0.1433	0.011388012	0.4259	3.89E-15
TARP	green	0.1423	0.012012734	0.4814	1.91E-19
TARP	green	0.1423	0.012012734	0.4814	1.91E-19
APOBEC3F	green	0.1411	0.012767643	0.4938	1.61E-20
HLA-DRB3	green	0.1387	0.014333322	0.5260	1.59E-23
RASGRP1	green	0.1382	0.014706632	0.5275	1.13E-23
HLA-B	green	0.1376	0.015172783	0.6527	3.89E-39
USP41	green	0.1369	0.015711123	0.4431	2.19E-16
RAMP2	green	0.1360	0.016382238	0.4382	5.04E-16
GBP3	green	0.1359	0.016517548	0.3837	2.39E-12
CXCL11	green	0.1358	0.01659341	0.4033	1.36E-13
FLT3LG	green	0.1349	0.017316007	0.4676	2.69E-18
KLRC4	green	0.1290	0.022923827	0.4429	2.25E-16
TNFSF10	green	0.1271	0.024969987	0.5320	3.98E-24
HLA-C	green	0.1271	0.02497374	0.6525	4.24E-39
ARHGAP25	green	0.1205	0.033695216	0.5125	3.17E-22
EEF1A1	green	0.1180	0.037502882	0.5215	4.35E-23
BTN2A2	green	0.1172	0.038814504	0.4472	1.06E-16
RNF213	green	0.1157	0.04137884	0.5632	1.98E-27
UBE2L6	green	0.1124	0.047607761	0.5151	1.79E-22
HLA-L	green	0.1124	0.047666456	0.4895	3.84E-20
HLA-L	green	0.1124	0.047666456	0.4895	3.84E-20
CMPK2	green	0.1124	0.047698506	0.3004	6.62E-08
PARP12	green	0.1120	0.048480556	0.5482	8.62E-26

EEF1A1	green	0.1065	0.060764443	0.5168	1.23E-22
PARP9	green	0.1053	0.06370653	0.5149	1.86E-22
ANGPTL6	green	0.1037	0.067921515	0.4248	4.69E-15
HLA-B	green	0.1036	0.068042398	0.6369	8.62E-37
ERAP2	green	0.1019	0.072863665	0.4140	2.60E-14
TNFSF13B	green	0.1017	0.073279182	0.5644	1.46E-27
TNFAIP2	green	0.1003	0.077422842	0.4441	1.83E-16
IRF9	green	0.0981	0.084268002	0.4977	7.20E-21
IDO1	green	0.0964	0.089674028	0.3661	2.69E-11
RNF213	green	0.0861	0.129605646	0.5581	7.26E-27
SLFN12L	green	0.0839	0.140018536	0.4546	2.89E-17
CIITA	green	0.0770	0.17579754	0.5769	5.55E-29
PSMB8	green	0.0594	0.296578986	0.4838	1.20E-19
PSMB8	green	0.0594	0.296578986	0.4838	1.20E-19
PSMB8	green	0.0594	0.296578986	0.4838	1.20E-19
SP110	green	0.0579	0.309113594	0.5540	2.06E-26
HLA-DQB2	green	0.0554	0.329980523	0.3342	1.50E-09
BST2	green	0.0536	0.346202098	0.4224	6.87E-15
GUSB	green	0.0497	0.381967936	0.3742	9.02E-12
HLA-DQB2	green	0.0493	0.386173353	0.4269	3.32E-15
HLA-DQB1	green	0.0474	0.404921382	0.4912	2.71E-20
HLA-DQB1	green	0.0474	0.404921382	0.4912	2.71E-20
GIMAP2	green	0.0448	0.431390019	0.5319	4.14E-24
SLFN5	green	0.0341	0.549173654	0.5658	1.04E-27
LAP3	green	0.0308	0.588041189	0.4036	1.31E-13
DYNC1I1	green	(0.0225)	0.693243626	(0.4131)	3.03E-14
GBP2	green	0.0211	0.711387625	0.5463	1.37E-25
IFI35	green	0.0186	0.743472218	0.3823	2.92E-12
DTX3L	green	(0.0148)	0.794933778	0.3820	3.04E-12
GLYR1	green	(0.0140)	0.805080994	(0.4039)	1.23E-13
HLA-B	green	0.0111	0.844917993	0.4878	5.36E-20
HLA-C	green	0.0007	0.99000263	0.4711	1.38E-18

Table.S3 The degree of node in PPI network.

Number	Gene name	Degree
1	STAT1	68
2	OAS1	63
3	OAS2	60
4	MX1	59
5	IRF9	58
6	CXCL10	56
7	OAS3	56
8	IFIT3	54
9	IFIH1	51
10	IFIT2	51
11	RSAD2	51
12	GBP2	50
13	MX2	49
14	TRIM22	48
15	B2M	46
16	DDX58	46
17	XAF1	45
18	HLA-A	44
19	STAT2	44
20	HLA-B	43
21	HLA-G	43
22	IFI35	43
23	VCAM1	43
24	PSMB8	42
25	USP18	42
26	TLR3	42
27	IFI44	42
28	HLA-C	41
29	HLA-F	41
30	CIITA	41
31	BST2	41
32	DDX60	41
33	CCL5	39
34	IFI6	39
35	IFI44L	39
36	GBP5	39
37	HLA-DRA	37
38	SAMD9L	37
39	RTP4	37
40	PARP9	36
41	DHX58	36
42	GBP4	35
43	PARP12	35
44	PSMB9	34
45	UBE2L6	34
46	HLA-DQA1	33
47	HLA-DPA1	33
48	HLA-DQB1	32
49	HLA-DPB1	32

50	EIF2AK2	32
51	CXCL11	32
52	GBP3	32
53	HLA-DQB2	31
54	CXCL9	31
55	CMPK2	31
56	PARP14	29
57	SP110	27
58	MAVS	24
59	CD74	24
60	UBA7	24
61	RNF213	24
62	CASP1	24
63	TNFSF10	24
64	SAMD9	23
65	CXCL12	21
66	HLA-DMA	20
67	CD3G	20
68	EPSTI1	20
69	HLA-DMB	19
70	HLA-DOA	19
71	IDO1	19
72	TNFSF13B	19
73	GBP7	19
74	NLRC5	18
75	CX3CR1	17
76	DTX3L	16
77	CD1C	14
78	IL2RB	14
79	CX3CL1	13
80	CTSK	12
81	LUM	10
82	ARRB1	10
83	DYNC1I1	10
84	ITGAL	10
85	FMOD	9
86	P2RY13	9
87	VCL	9
88	NT5E	9
89	CD83	9
90	ASPN	9
91	FLT3LG	9
92	APLNR	8
93	APOBEC3G	8
94	P2RX7	8
95	BCL2L1	8
96	TIMP2	7
97	COL12A1	7
98	KLRC4	7
99	OGN	6
100	COL14A1	6
101	PLCB1	6
102	LAP3	6
103	DPT	6

104	OMD	5
105	NR3C1	5
106	PLCG2	5
107	ACKR4	5
108	DDX60L	5
109	ACE	5
110	COL15A1	4
111	STON2	4
112	HIP1R	4
113	ISLR	4
114	SSPN	4
115	PRDM1	4
116	IL34	4
117	PARP8	4
118	GPR174	4
119	RECK	3
120	GNA14	3
121	GUSB	3
122	LPAR4	3
123	APOBEC3F	3
124	PPP3CC	3
125	EML4	3
126	LPXN	3
127	RGS4	3
128	MFAP4	3
129	SULF1	3
130	BICC1	3
131	STAMBPL1	3
132	USP2	3
133	ITGBL1	3
134	ERAP2	3
135	MXRA5	3
136	BTN3A2	2
137	BTN3A1	2
138	RNASET2	2
139	ESRRA	2
140	NRN1	2
141	NTM	2
142	MARK3	2
143	PRDX6	2
144	LRMP	2
145	BTN3A3	2
146	SFRP4	2
147	ENPP2	2
148	EEF1A1	2
149	FRZB	2
150	PARP15	2
151	PDE5A	2
152	PCNT	2
153	MTSS1L	2
154	KCNN3	2
155	SLFN12L	2
156	APOBEC3D	2
157	ADAM22	2

158	IL18R1	2
159	CLEC4A	2
160	TCF4	2
161	KCNJ11	2
162	PI16	2
163	FCER1A	2
164	GPR124	2
165	SLC29A2	2
166	INMT	1
167	MAGED2	1
168	LCN10	1
169	LCN6	1
170	SNTB1	1
171	LIMA1	1
172	HAAO	1
173	PLA2R1	1
174	MYOF	1
175	KCNA3	1
176	EXT1	1
177	SAMD3	1
178	MYH10	1
179	CRHBP	1
180	RASGRP1	1
181	ALDH1A2	1
182	TRIL	1
183	APOL6	1
184	PYHIN1	1
185	SPATA18	1
186	FRMD4B	1
187	EPHA3	1
188	GIMAP2	1
189	SNCAIP	1
190	ZNF423	1
191	TMTC1	1
192	LIPH	1
193	ANTXR1	1
194	HTRA1	1
195	SLCO4A1	1
196	NR2F6	1
197	IL1RL1	1
198	MARCKS	1
199	HMCN1	1

Table.S4 The SAM ananysis result of ISCM-related significant genes.						
**probe**	**genesymbol**	**expected score**	**Observed score**	**numerator**	**denominator(s+s0)**	**Fold change**
8101675	ABCG2	0.4374	10.2600	0.7437	0.0725	1.6521
8122334	ACKR4	0.6113	10.2632	1.0868	0.1059	2.1281
7955562	ACVRL1	-0.6381	-9.7523	-0.4457	0.0457	0.7298
8088560	ADAMTS9	0.3422	-10.8345	-1.1480	0.1060	0.4222
8092457	ALG3	0.3684	-9.5876	-0.4681	0.0488	0.7100
8174576	AMOT	1.4465	9.0035	0.4722	0.0525	1.3913
7929653	ANKRD2	-0.9461	-9.3338	-1.1464	0.1228	0.4958
8022559	ANKRD29	-0.1266	9.3220	0.6178	0.0663	1.5131
7985238	ANKRD34C	-0.3925	9.0525	0.5340	0.0590	1.4847
8054054	ANKRD36	0.1022	9.3884	0.4734	0.0504	1.3880
8043697	ANKRD36B	0.0375	9.9982	0.4832	0.0483	1.3946
8053801	ANKRD36C	0.1006	9.7364	0.4940	0.0507	1.4036
8171248	ANOS1	1.3405	11.0192	0.7781	0.0706	1.6300
8042439	ANTXR1	0.0281	9.0899	0.5936	0.0653	1.5776
8146159	AP3M2	0.8677	10.7843	0.4919	0.0456	1.3960
8114593	APBB3	0.5392	11.0098	0.5455	0.0495	1.4365
8160670	AQP3	1.0861	-9.9344	-0.8668	0.0872	0.5265
7947681	ARHGAP1	-0.7238	10.7344	0.4827	0.0450	1.3959
8113073	ARRDC3	0.5273	11.0262	0.7646	0.0693	1.6907
8162394	ASPN	1.1208	12.9776	1.7725	0.1366	2.8148
7904254	ATP1A1	-1.4951	-9.9338	-0.5423	0.0546	0.6676
8015955	ATXN7L3	-0.1731	-9.1747	-0.3599	0.0392	0.7755
8065569	BCL2L1	0.1746	-9.0394	-0.4594	0.0508	0.7185
8092691	BCL6	0.3705	-10.5357	-0.6712	0.0637	0.6246
7952116	BCL9L	-0.6728	-9.6175	-0.5112	0.0532	0.6825
8074261	BID	0.2410	-9.1390	-0.4066	0.0445	0.7516
8117458	BTN3A1	0.5660	12.1095	0.7089	0.0585	1.6195
7951521	C11orf65	-0.6786	10.1737	0.4199	0.0413	1.3459
7990269	C15orf59	-0.3611	-10.5163	-0.7416	0.0705	0.5764
7999291	C16orf89	-0.2945	10.1347	0.6638	0.0655	1.6360
8036867	C19orf47	-0.0163	-9.1705	-0.4106	0.0448	0.7597

7903980	C1orf162	-1.5058	-9.9121	-0.8693	0.0877	0.5587
8010287	C1QTNF1	-0.2136	-10.7350	-1.0027	0.0934	0.4670
8115594	C1QTNF2	0.5474	9.7325	0.5474	0.0562	1.4801
8094184	C1QTNF7	0.3824	11.3941	0.6126	0.0538	1.5602
7996198	CCDC113	-0.3176	11.3518	0.7869	0.0693	1.6714
8177628	CCDC125	1.6064	8.9942	0.3931	0.0437	1.3068
7972670	CCDC168	-0.4986	9.4148	0.5979	0.0635	1.5373
8154416	CCDC171	0.9834	9.2206	0.4278	0.0464	1.3375
7942123	CCND1	-0.7891	10.1005	0.4935	0.0489	1.3995
7957052	CCT2	-0.6228	-9.1314	-0.5709	0.0625	0.6509
7960794	CD163	-0.5920	-12.1795	-1.4449	0.1186	0.4171
7934334	CFAP70	-0.8909	10.3474	0.4695	0.0454	1.3852
8025918	CNN1	-0.1023	-9.2547	-0.9843	0.1064	0.4974
8148070	COL14A1	0.8898	11.6109	1.3038	0.1123	2.5760
8147469	CPQ	0.8835	10.3810	0.4922	0.0474	1.4056
8131996	CREB5	0.7039	10.0227	0.6818	0.0680	1.5606
8146967	CRISPLD1	0.8778	11.2325	0.9538	0.0849	2.2184
8073449	CSDC2	0.2346	-12.6490	-0.8752	0.0692	0.5559
7919815	CTSK	-1.0967	9.3329	0.8243	0.0883	1.9722
8103563	DDX60	0.4527	9.1725	0.5111	0.0557	1.4150
7934533	DUSP13	-0.8887	-9.2073	-0.5917	0.0643	0.6509
8081503	DZIP3	0.2934	9.9122	0.5457	0.0551	1.4271
8162404	ECM2	1.1210	13.6895	1.0054	0.0734	2.0405
7973108	ECRP	-0.4928	-10.1003	-0.8719	0.0863	0.4966
8061428	ENTPD6	0.1450	-9.0389	-0.3960	0.0438	0.7569
8145532	EPHX2	0.8609	9.4186	0.7333	0.0779	1.6404
8031550	EPN1	-0.0574	-9.1092	-0.3664	0.0402	0.7687
7923596	ETNK2	-1.0310	-9.2699	-0.8068	0.0870	0.5135
8152491	EXT1	0.9571	9.3436	0.4933	0.0528	1.4018
7914015	FAM46B	-1.2043	-9.9496	-0.7422	0.0746	0.5831
8175811	FAM58A	1.5113	-9.5133	-0.5077	0.0534	0.6934
7967879	FAM58DP	-0.5347	-9.1903	-0.4518	0.0492	0.7183
8078461	FBXL2	0.2734	9.7358	0.4813	0.0494	1.3942
7914075	FCN3	-1.2034	-17.6990	-1.9586	0.1107	0.2444
7972650	FGF14	-0.4988	9.6767	0.7014	0.0725	1.5958

8013191	FLII	-0.1943	-10.0589	-0.3921	0.0390	0.7589
7907297	FMO4	-1.3700	9.1841	0.6932	0.0755	1.5637
8123104	FNDC1	0.6174	13.4989	1.0367	0.0768	2.0889
8160168	FREM1	1.0748	16.0401	1.0409	0.0649	2.0594
8057506	FRZB	0.1205	13.6377	1.3123	0.0962	2.5051
7986092	FURIN	-0.3860	-12.2237	-0.5984	0.0490	0.6475
8157976	GARNL3	1.0398	9.3390	0.5816	0.0623	1.4816
8074991	GGT5	0.2477	-12.9118	-0.8169	0.0633	0.5679
7965941	GLT8D2	-0.5486	11.0480	0.8072	0.0731	1.6852
8037614	GPR4	-0.0098	-10.9245	-0.6761	0.0619	0.6124
8168968	GPRASP1	1.2684	9.0021	0.5502	0.0611	1.4526
8096335	HERC6	0.3988	9.1780	0.6673	0.0727	1.5475
7909510	HHAT	-1.3148	9.3085	0.4291	0.0461	1.3380
8124524	HIST1H2AK	0.6321	10.0216	0.5383	0.0537	1.4457
8091354	HLTF	0.3603	11.6427	0.5960	0.0512	1.4800
8000409	HMGN2	-0.2869	14.3005	0.7011	0.0490	1.5931
7982204	HMGN2	-0.4144	14.3770	0.7029	0.0489	1.5955
7992987	HMOX2	-0.3389	-15.0375	-0.6812	0.0453	0.6196
7898677	HS6ST1	-1.8711	-9.1654	-0.4734	0.0516	0.7078
7931097	HTRA1	-0.9289	11.6050	0.5744	0.0495	1.5026
8129985	HYMAI	0.6836	8.9933	0.4314	0.0480	1.3531
7902541	IFI44L	-1.5715	11.7185	1.1645	0.0994	2.2196
7929047	IFIT2	-0.9540	9.7996	0.6300	0.0643	1.5908
7929052	IFIT3	-0.9538	9.2106	0.5721	0.0621	1.5343
8044035	IL18R1	0.0395	-9.0797	-0.6552	0.0722	0.6196
8044021	IL1RL1	0.0394	-12.6394	-1.8058	0.1429	0.2173
8097307	INTU	0.4049	10.2051	0.3999	0.0392	1.3173
7984813	ISLR	-0.3964	12.6437	0.8595	0.0680	1.8240
7931977	ITIH5	-0.9182	12.2380	0.8022	0.0655	1.7411
7980970	ITPK1	-0.4309	-10.4801	-0.4161	0.0397	0.7457
7920409	JTB	-1.0832	-9.4167	-0.3598	0.0382	0.7751
7920552	KCNN3	-1.0812	12.1503	0.7044	0.0580	1.6238
7922474	KIAA0040	-1.0468	-9.3068	-0.6234	0.0670	0.6442
8099817	KLF3-AS1	0.4234	9.8671	0.4466	0.0453	1.3691
8174654	KLHL13	1.4490	9.2936	0.6519	0.0701	1.5335

7923347	LAD1	-1.0353	-13.7569	-0.6980	0.0507	0.6142
8128991	LAMA4	0.6749	9.6049	0.5518	0.0574	1.4484
7990080	LARP6	-0.3622	-9.5206	-0.4204	0.0442	0.7439
8165271	LCN10	1.1767	-10.1583	-0.5614	0.0553	0.6659
8165277	LCN6	1.1770	-12.4691	-0.8907	0.0714	0.5198
8005043	LINC00670	-0.2530	9.2249	0.8154	0.0884	1.7202
8056983	LOC100130691	0.1192	9.2580	0.4055	0.0438	1.3183
8086183	GOLGA4	0.3262	9.2130	0.4856	0.0527	1.3905
8168517	LPAR4	1.2563	9.5248	0.5392	0.0566	1.4640
7960730	LPCAT3	-0.5926	-10.0918	-0.6179	0.0612	0.6461
8135218	LRRC17	0.7366	9.5539	0.9262	0.0969	1.8864
8028872	LTBP4	-0.0799	10.9012	0.5742	0.0527	1.4875
7965403	LUM	-0.5528	13.0900	1.2522	0.0957	2.1768
7946579	LYVE1	-0.7352	-10.6769	-1.3458	0.1260	0.4418
7914042	MAP3K6	-1.2037	-9.0477	-0.4996	0.0552	0.7068
7977077	MARK3	-0.4606	-9.3455	-0.4755	0.0509	0.7062
8147516	MATN2	0.8840	12.1714	0.8797	0.0723	1.8134
8058462	MDH1B	0.1253	9.5906	0.4874	0.0508	1.4086
8013341	MFAP4	-0.1927	10.5009	0.9137	0.0870	1.8736
8083494	MME	0.3074	10.2072	0.9503	0.0931	2.1244
7989146	MNS1	-0.3678	15.5730	1.0887	0.0699	2.0499
8126066	MTCH1	0.6486	-10.3480	-0.3717	0.0359	0.7689
8171172	MXRA5	1.3380	9.9160	1.2280	0.1238	2.1242
8012475	MYH10	-0.1989	9.3768	0.5596	0.0597	1.4861
7977987	MYH6	-0.4526	-11.4961	-1.6099	0.1400	0.3711
8108256	MYOT	0.4871	-10.1479	-1.1589	0.1142	0.4192
8175924	NAA10	1.5151	-10.1572	-0.3545	0.0349	0.7770
8173917	NAP1L3	1.4198	9.7585	0.9644	0.0988	1.8478
8072242	NF2	0.2261	-9.1045	-0.4461	0.0490	0.7155
7968703	NHLRC3	-0.5285	9.9457	0.4487	0.0451	1.3679
7925320	NID1	-1.0066	-10.0389	-0.6081	0.0606	0.6483
8134463	NPTX2	0.7290	-11.3567	-0.8386	0.0738	0.5344
8169115	NRK	1.2743	12.1123	0.9363	0.0773	1.8894
8087935	NT5DC2	0.3379	-11.5726	-0.6520	0.0563	0.6307
8120967	NT5E	0.5981	9.6318	0.7301	0.0758	1.7446

7945245	NTM	-0.7544	9.9591	0.6746	0.0677	1.6979
8162373	OGN	1.1203	12.7874	1.4122	0.1104	2.4801
7938225	OLFML1	-0.8413	10.6136	0.9360	0.0882	1.8989
7913883	PAFAH2	-1.2073	9.0437	0.4297	0.0475	1.3413
8102532	PDE5A	0.4446	12.6124	0.9560	0.0758	1.8997
7965040	PHLDA1	-0.5556	11.4513	1.0983	0.0959	2.0814
8158431	PHYHD1	1.0461	-10.0889	-0.5291	0.0524	0.6878
8119124	PI16	0.5836	9.0843	1.0020	0.1103	1.9784
7949808	PITPNM1	-0.6975	-10.0636	-0.4775	0.0474	0.7195
7913216	PLA2G2A	-1.2200	-10.7190	-1.8040	0.1683	0.3898
8041644	PLEKHH2	0.0227	10.7329	0.9023	0.0841	1.9364
8167449	PLP2	1.2252	-9.2867	-0.6229	0.0671	0.6303
8128394	PNISR	0.6700	9.2370	0.3439	0.0372	1.2578
8102567	PRDM5	0.4448	9.5845	0.4722	0.0493	1.3825
8146794	PREX2	0.8761	9.2330	0.4150	0.0450	1.3385
8143144	PTN	0.8297	12.5136	1.0304	0.0823	2.0441
7914489	PTP4A2	-1.1943	-9.3286	-0.3594	0.0385	0.7736
7907830	QSOX1	-1.3578	-9.0527	-0.4958	0.0548	0.7020
7989670	RBPMS2	-0.3652	-9.3325	-0.4752	0.0509	0.7312
7973110	RNASE2	-0.4927	-11.4702	-1.1786	0.1028	0.4087
8156278	S1PR3	1.0123	-11.8018	-0.5944	0.0504	0.6560
7952011	SCN2B	-0.6738	10.9745	0.8198	0.0747	1.7208
7946454	SCUBE2	-0.7360	12.2534	0.8719	0.0712	1.8521
7959002	SDSL	-0.6074	11.2524	0.6799	0.0604	1.5906
7985934	SEMA4B	-0.3869	-10.8262	-0.6806	0.0629	0.6060
8032839	SEMA6B	-0.0459	-9.2194	-0.5016	0.0544	0.6917
7976496	SERPINA3	-0.4671	-18.8601	-2.5850	0.1371	0.1864
8150428	SFRP1	0.9293	9.0080	1.0603	0.1177	1.9337
8139087	SFRP4	0.7811	13.4721	1.7946	0.1332	4.5515
7933750	SLC16A9	-0.8976	11.6713	0.8411	0.0721	1.8135
7950990	SLC36A4	-0.6848	-9.1395	-0.6102	0.0668	0.6326
7920401	SLC39A1	-1.0835	-9.0469	-0.3515	0.0389	0.7782
8063923	SLCO4A1	0.1627	-12.9103	-1.2684	0.0982	0.4309
8123446	SMOC2	0.6202	14.5535	1.1154	0.0766	2.1346
8101762	SNCA	0.4381	9.1559	0.6640	0.0725	1.5941

7954481	SSPN	-0.6471	9.1213	0.4729	0.0519	1.3630
7902425	ST6GALNAC3	-1.5787	-10.1212	-0.6275	0.0620	0.6433
8180309	ST6GALNAC3	2.0109	-9.6082	-0.6613	0.0688	0.6306
8015607	STAT3	-0.1756	-9.6980	-0.5688	0.0587	0.6589
8038347	TEAD2	-0.0035	-10.5722	-0.4589	0.0434	0.7208
7953150	TEAD4	-0.6609	-9.9392	-0.6770	0.0681	0.6014
8075239	THOC5	0.2497	-9.2865	-0.3955	0.0426	0.7497
7899627	TINAGL1	-1.7551	-9.1323	-0.4278	0.0468	0.7441
7935296	TLL2	-0.8801	10.9121	0.9241	0.0847	1.8976
8152976	TMEM71	0.9633	9.2427	0.8842	0.0957	1.7744
8066964	TMEM189	0.1846	-9.7296	-0.3617	0.0372	0.7757
7962058	TMTC1	-0.5795	-9.9740	-0.5361	0.0537	0.6869
8143879	TMUB1	0.8386	-9.5079	-0.3713	0.0391	0.7705
8075106	TPST2	0.2486	-9.5044	-0.4012	0.0422	0.7493
8068522	TTC3	0.1972	10.4499	0.3984	0.0381	1.3133
8077612	TTLL3	0.2675	9.4535	0.4436	0.0469	1.3554
8045291	TUBA3D	0.0478	-14.8910	-0.9564	0.0642	0.5098
8055194	TUBA3E	0.1098	-11.2194	-0.8044	0.0717	0.5631
8087485	UBA7	0.3345	9.1188	0.4803	0.0527	1.3884
8000244	USP31	-0.2878	-10.5416	-0.5784	0.0549	0.6591
8173287	VSIG4	1.4003	-11.9323	-1.3062	0.1095	0.4140
8173059	WNK3	1.3914	-9.4907	-0.6998	0.0737	0.5915
8004184	XAF1	-0.2585	9.8137	0.5592	0.0570	1.4760
7929634	ZDHHC16	-0.9465	-10.4408	-0.4570	0.0438	0.7204
8174119	ZMAT1	1.4275	12.6971	0.7155	0.0564	1.5943
8157193	ZNF483	1.0263	9.1079	0.5531	0.0607	1.4358
8025984	ZNF844	-0.1014	9.0949	0.5094	0.0560	1.3990

Table S5. Top 20 clusters of Pathway and Process Enrichment Analysis

No.	GO	Category	Description	Count	Inquired genes %	Log10(P)	Log10(q)
1	R-HSA-913531	Reactome Gene Sets	Interferon Signaling	42	15.67	-40.71	-36.4
2	GO:0051607	GO Biological Processes	defense response to virus	35	13.06	-27.6	-24.1
3	GO:0001817	GO Biological Processes	regulation of cytokine production	44	16.42	-18.83	-15.68
4	hsa04612	KEGG Pathway	Antigen processing and presentation	18	6.72	-18.48	-15.36
5	GO:0045088	GO Biological Processes	regulation of innate immune response	25	9.33	-10.31	-7.61
6	GO:0002697	GO Biological Processes	regulation of immune effector process	25	9.33	-10.01	-7.36
7	GO:0042110	GO Biological Processes	T cell activation	25	9.33	-9.89	-7.25
8	GO:0002480	GO Biological Processes	antigen processing and presentation of exogenous peptide antigen via MHC class I, TAP-independent	6	2.24	-9.84	-7.23
9	R-HSA-1169410	Reactome Gene Sets	Antiviral mechanism by IFN-stimulated genes	11	4.1	-8.81	-6.29
10	GO:0035455	GO Biological Processes	response to interferon-alpha	6	2.24	-7.08	-4.73
11	M5885	Canonical Pathways	NABA MATRISOME ASSOCIATED	27	10.07	-7.01	-4.67
12	GO:0009617	GO Biological Processes	response to bacterium	26	9.7	-6.72	-4.41
13	GO:0060760	GO Biological Processes	positive regulation of response to cytokine stimulus	8	2.99	-6.67	-4.36
14	GO:0002683	GO Biological Processes	negative regulation of immune system process	20	7.46	-6.27	-3.99
15	R-HSA-3656225	Reactome Gene Sets	Defective CHST6 causes MCDC1	4	1.49	-6	-3.74
16	GO:0002399	GO Biological Processes	MHC class II protein complex assembly	3	1.12	-5.87	-3.64
17	CORUM:60	CORUM	Interferon-stimulated gene factor 3 transcription comple	3	1.12	-5.87	-3.64
18	R-HSA-8983711	Reactome Gene Sets	OAS antiviral response	4	1.49	-5.75	-3.54
19	GO:0030198	GO Biological Processes	extracellular matrix organization	16	5.97	-5.32	-3.17
20	M54	Canonical Pathways	PID IL12 2PATHWAY	7	2.61	-5.21	-3.07

Table S6. The list of eleven candidate genes

Number	GeneSymbol	Degree	Fold change
1	IFIT3	54	1.53
2	IFIT2	51	1.59
3	XAF1	45	1.48
4	DDX60	41	1.42
5	IFI44L	39	2.22
6	UBA7	24	1.39
7	CTSK	12	1.97
8	LUM	10	2.18
9	NT5E	9	1.74
10	ASPN	9	2.81
11	BCL2L1	8	0.72

The candidate genes, hub genes, satisfies both the degree value greater than or equal to 8 and the fold change more than 1.2 times.

Table S7. The Primer informtion of hub genes for RT-qPCR in Sprague Dawley rat hearts.

Number	Gene ID	GeneSymbol	Forward / Reverse Primer	Sequence	Location	TM(℃)	Product(bp)
1	306805	ASPN	Forward Primer	ACCTCCATCCCGAAAGGACTA	888-908	57.6	128
			Reverse Primer	TTGTTTCCAAGACCCAGCCT	1015-996	56.8	
2	24888	BCL2L1	Forward Primer	TCGCTAAACACAGAGCAGACC	85-105	57.3	97
			Reverse Primer	CCGGTTGCTCTGAGACATTTTTA	181-159	55	
3	29175	CTSK	Forward Primer	CGTATGTGGGGCAGGATGAAA	678-698	57.6	138
			Reverse Primer	ACACAGAGACGGGTCCTACC	815-796	59.1	
4	100360801	DDX60	Forward Primer	AGAAGAGGGCAACGTTCTAGC	5454-5474	57.2	197
			Reverse Primer	GGAGGTCATCAAGGAACACCTTT	5650-5628	57	
5	310968	IFI44L	Forward Primer	GCATCACCACGCAGTACAAAG	880-900	57	94
			Reverse Primer	TCCTCCAGCCCCATTGAATC	973-954	57.4	
6	294091	IFIT2	Forward Primer	GACACAGCAGTTGAGTGTGC	146-165	57	91
			Reverse Primer	ATGATTCCTTACTGGCTGTACTCA	236-213	55	
7	309526	IFIT3	Forward Primer	ATCGTCTGAGTGCCCACTTT	59-78	56.4	115
			Reverse Primer	ACTCCTTGTTGACCTCACTCAT	173-152	55.5	
8	81682	LUM	Forward Primer	GTGTCAAGAGAGTAAGGGCACA	930~1020	56.8	159
			Reverse Primer	CATTCTGGTGCACAGTTGGG	167~148	57	
9	58813	NT5E	Forward Primer	TCCTGCAAGTGGGTGGAATC	1387-1406	57.8	178
			Reverse Primer	AATCCATCCCCACCGTTGAC	1564-1545	57.8	
10	301000	UBA7	Forward Primer	CCTGTCCGTGTGCAGAAAGAT	773-793	57.7	206
			Reverse Primer	TGGAAGGTCTGGTGTAGGCA	978-959	58.5	
11	679600	XAF1	Forward Primer	TGGCACAGCAGAAGACTAGC	95-114	57.6	247
			Reverse Primer	GGCCACACTTCTTTTGCAGTT	341-321	56.3	
12	24383	GAPDH	Forward Primer	GTATCGGACGCCTGGTTAC	107-125	55.7	128
			Reverse Primer	CTTGCCGTGGGTAGAGTCAT	234-215	57.2	

Table S8 The variantS associations with coronary artery disease

Gene	nParam	pValue	Disease Phenotype	subjects	zStat	chromosome	start	end	type
IFI44L	10	0.0023	Myocardial infarction	20119	2.8336	1	79085607	79111830	protein_coding
IFI44L	1	0.0186	Open phenotype page	44456	2.0837	1	79085607	79111830	protein_coding
IFI44L	25	0.0238	Open region page with selected phenotype	56637	1.9802	1	79085607	79111830	protein_coding
IFI44L	16	0.0277	P-wave duration	1072402	1.9158	1	79085607	79111830	protein_coding
IFI44L	6	0.0311	Any cardiovascular	300	1.8645	1	79085607	79111830	protein_coding
IFI44L	19	0.0338	Coronary artery disease	2926	1.8273	1	79085607	79111830	protein_coding
IFI44L	5	0.0373	Coronary heart disease or stroke or peripheral vascular disease in type 2	83462	1.7834	1	79085607	79111830	protein_coding
IFI44L	6	0.0461	Obese vs thin	106080	1.6842	1	79085607	79111830	protein_coding
IFIT2	7	0.0006	QRS interval	1071907	3.2510	10	91043451	91069028	protein_coding
IFIT2	14	0.0009	Creatine kinase	300871	3.1158	10	91043451	91069028	protein_coding
IFIT2	14	0.0022	Weight	14234	2.8499	10	91043451	91069028	protein_coding
IFIT2	10	0.0032	Dyslipidemia	56375	2.7249	10	91043451	91069028	protein_coding
IFIT2	3	0.0033	Fasting plasma free fatty	9584	2.7123	10	91043451	91069028	protein_coding
IFIT2	10	0.0209	Hypertension	70356	2.0346	10	91043451	91069028	protein_coding
IFIT2	2	0.0272	QRS interval	78778	1.9242	10	91043451	91069028	protein_coding
IFIT2	14	0.0399	Systolic blood pressure	300378	1.7519	10	91043451	91069028	protein_coding
IFIT3	17	0.0002	Diastolic blood pressure	385253	3.5453	10	91087754	91100728	protein_coding
IFIT3	12	0.0005	Coronary artery disease	961788	3.2951	10	91087754	91100728	protein_coding
IFIT3	17	0.0136	Systolic blood pressure	384710	2.2088	10	91087754	91100728	protein_coding
IFIT3	8	0.0160	HDL3 cholesterol	10984	2.1452	10	91087754	91100728	protein_coding
IFIT3	6	0.0193	Coronary artery disease in subjects without	212373	2.0688	10	91087754	91100728	protein_coding
IFIT3	1	0.0388	P-wave duration	44456	1.7653	10	91087754	91100728	protein_coding
IFIT3	15	0.0422	Dyslipidemia	56375	1.7260	10	91087754	91100728	protein_coding
IFIT3	8	0.0445	HDL2 cholesterol	10984	1.7008	10	91087754	91100728	protein_coding
