# Supplementary material for: Identified Three Interferon Induced Proteins as Novel Biomarkers of Human Ischemic Cardiomyopathy
Source: Int J Mol Sci. 2021 Dec 4;22(23):13116. doi: 10.3390/ijms222313116 (PMC8657967; doi:10.3390/ijms222313116)
Supplement: Supplementary file 1 [file ijms-22-13116-s001.zip › Supplementary material legend.pdf]

Identified interferon induced proteins as novel potential biomarkers significantly associated with human ischemic cardiomyopathy

Cheng Chen [1,2,3], Jiao Tian [1,4], Zhicheng He [1,2], Wenyong Xiong [1], Yingying He [5,*], Shubai Liu [1,2,*]

[1] State Key Laboratory of Phytochemistry and Plant Resources in West China, Kunming Institute of Botany, Chinese Academy of Sciences, Kunming 650201, China; Chencheng@mail.kib.ac.cn (C.C.); hezhicheng@mail.kib.ac.cn (Z.H.); wenyong.xiong@mail.kib.ac.cn (W.X.)

[2] University of Chinese Academy of Sciences, Beijing 100049, China;

[3] Department of pharmacy, the First People's Hospital of Yunnan, the affiliated Hospital of Kunming University of Science & Technology, Kunming, 650032, Yunnan, China; Chencheng@mail.kib.ac.cn (C.C.);

4 School of Life Sciences, Yunnan University, Kunming 650091, China; tianjiao@mail.kib.ac.cn;

5 School of Chemical Science & Technology, Yunnan University, Kunming 650091, China; yingying.he10@gmail.com (Y.H.);

* Correspondence: liushubai@mail.kib.ac.cn (S.L.); Tel.: +86-871-6522-3309 (S.L.,)

Correspondence to:

Dr. Shubai Liu,

Mailing address: State Key Laboratory of Phytochemistry and Plant Resources in West China, Kunming Institute of Botany, Chinese Academy of Sciences, 132 Blue Black Street, Kunming, Yunnan 650201, China; Phone: (86)871-65223309; E-mail: liushubai@mail.kib.ac.cn.

Supplementary index:

Figure S1. The sample dendrogram and trait heatmap.

Figure S2. The scatterplots of Gene Significance (GS) for histology vs. Module Membership (MM) in the significant modules (A~C).

Figure S3. The heart rate and cardiac function after administration metoprolol for 3 days.

Table S1. The biopsy samples information of expression profile.

Table S2. The significance genes related to ISCM feature.

Table S3. The degree of node in PPI network.

Table S4. The SAM analysis result of ISCM-related significant genes.

Table S5. Top 20 clusters of Pathway and Process Enriched of ischemic cardiomyopathy related significance genes.

Table S6. The list of eleven candidate genes.

Table S7. The Primer information of hub genes for RT-qPCR.

Table S8. The variants associations with coronary artery disease.

Supplementary Figure S1. The sample dendrogram and trait heatmap. The distinct co-expression modules were identified that significantly related to different pathological features (A). Clustering dendrogram of samples based on their Euclidean distance. The clinical feature traits were heart failure, IDCM and ISCM, gender and age. IDCM: Idiopathic Di1ated Cardiomyopathy; ISCM: ischemic cardiomyopathy. The white color means a low value, red means a high value. There are three case groups were separated by performed hierarchical clustering, were heart failure, IDCM and ISCM, respectively. (B) The correlation of Module-clinical traits. Each row corresponds to a module; each column corresponds to a clinical trait feature. Each cell contains the test statistic value and its corresponding p value from the linear mixed-effects model. Network of eigengene represents the relationships among the modules and the histological traits. There are fourteen modules were detected through the dataset. Four modules were significantly positive correlated to ISCM pathological feature, including tan (t-value = 0.18, p-value = 0.001), blue (t-value = 0.31, p-value = 4e-08), pink (t-value = 0.22, p-value = 1e-04), green (t-value = 0.4, p-value = 2e-13). The green module was the most significant correlation to ischemic cardiomyopathy.

Supplementary Figure S2. The scatterplots of Gene Significance (GS) for histology vs. Module Membership (MM) in the significant modules (A~C).

There is a highly significant correlation between GS and MM in this module, implying that the most important (central) elements of blue module also tend to be highly correlated with ISCM pathological trait. The green module (t-value = 0.59, p-value = 1.1e−31) has the highest correlation with ischemic cardiomyopathy status. It suggested that these gene contained in green module were significant associated with ischemic cardiomyopathy.

Supplementary Figure S3. The heart rate and cardiac function after administration metoprolol for 3 days. The heart rate of rat in Sham group and LAD group were 346.2 ± 4.664 (n=10) and 369.8 ± 13.44 (n=8), respectively (A). Treatment with metoprolol decreased the rat's heart rate to 321.0 ± 8.595 (**P<0.01, n=10). Evaluation methods of cardiac function (B). LVEF: left ventricular ejection fraction; LVFS: left ventricular fractional shortening; IVSs, interventricular septal thickness in systole; LVIDs: left ventricular internal diameter in systole; LVPWs: LV posterior wall thickness in systole; IVSd: interventricular septal thickness in diastole; LVIDd: left ventricular internal dimension-diastole; LVPWd: LV posterior wall thickness in diastole (C)-(H). Results were expressed as the means±SEM and a dependent t-test would be used. **P<0.001 and ****P<0.0001. Sham group, n=10; Lad group, n=8; MET group, n=10.

Supplementary Table S1. The biopsy samples information of expression profile.

Supplementary Table S2. The significance genes related to ISCM feature.

Supplementary Table S3. The degree of node in PPI network.

Supplementary Table S4. The SAM analysis result of ISCM-related significant genes.

Supplementary Table S5. Top 20 clusters of Pathway and Process Enrichment Analysis

Supplementary Table S6. The list of eleven candidate genes.

Supplementary Table S7. The Primer information of hub genes for RT-qPCR.

Supplementary Table S8. The variants associations with coronary artery disease.
